# Cue-Polarized Transport of β-actin mRNA Depends on 3′UTR and Microtubules in Live Growth Cones

**DOI:** 10.3389/fncel.2018.00300

**Published:** 2018-09-10

**Authors:** Kin-Mei Leung, Bo Lu, Hovy Ho-Wai Wong, Julie Qiaojin Lin, Benita Turner-Bridger, Christine E. Holt

**Affiliations:** Department of Physiology, Development and Neuroscience, University of Cambridge, Cambridge, United Kingdom

**Keywords:** axon navigation, growth cone, mRNA trafficking, β-actin, 3′UTR, local protein synthesis, translation

## Abstract

Guidance cues trigger fast responses in axonal growth cones such as directional turning and collapse that require local protein synthesis. An attractive cue-gradient, such as Netrin-1, triggers *de novo* synthesis of β-actin localized to the near-side compartment of the growth cone that promotes F-actin assembly and attractive steering. How this precise spatial asymmetry in mRNA translation arises across the small expanse of the growth cone is poorly understood. Pre-localized mRNAs in the vicinity of activated receptors could be selectively translated and/or new mRNAs could be trafficked into the area. Here we have performed live imaging of fluorescent-tagged β-actin mRNA to investigate mRNA trafficking dynamics in *Xenopus* retinal ganglion cell (RGC) axons and growth cones in response to Netrin-1. A Netrin-1 gradient was found to elicit the transport of β-actin mRNA granules to the near-side of growth cones within a 4–7 min window. This polarized mRNA trafficking depended on the 3′ untranslated region (UTR) since mRNA-Δ3′UTR mutant failed to exhibit cue-induced localization. Global application of Netrin-1 significantly increased the anterograde movement of β-actin mRNA along axons and also promoted microtubule-dependent mRNA excursions from the central domain of the growth cone into the periphery (filopodia and lamellipodia). Dual channel imaging revealed β-actin mRNA riding behind the microtubule plus-end tracking protein, EB1, in movements along dynamic microtubules into filopodia. The mRNA-EB1 movements were unchanged by a Netrin-1 gradient indicating the dynamic microtubules themselves do not underlie the cue-induced polarity of RNA movement. Finally, fast-moving elongated “worm-like” trains of Cy3-RNA, distinct from mitochondria, were seen transporting RNA along axons *in vitro* and *in vivo* suggesting the existence of a novel transport organelle. Overall, the results provide evidence that the axonal trafficking of β-actin mRNA can be regulated by the guidance cue Netrin-1 to transduce the polarity of an extracellular stimulus and that the 3′UTR is essential for this cue-induced regulation.

## Introduction

Local protein synthesis can be elicited rapidly by extrinsic cues and is required in axons for a wide range of specialized activities such as directional turning, branching, maintenance, and repair (Campbell and Holt, 2001; Verma et al., 2005; Wu et al., 2005; Leung et al., 2006; Piper et al., 2006; Yao et al., 2006; Cox et al., 2008; Spillane et al., 2012; Yoon et al., 2012; Cosker et al., 2013; Donnelly et al., 2013; Wong et al., 2017; Cagnetta et al., 2018; Terenzio et al., 2018). Growth cone turning toward an attractive gradient of Netrin-1, brain derived neurotrophic factor (BDNF) or Sonic Hedgehog (SHH) requires the *asymmetric* synthesis of β-actin protein on the near-side of the growth cone, closest to the source (Leung et al., 2006; Yao et al., 2006; Lepelletier et al., 2017), facilitating cytoskeletal assembly in the direction of growth. The mechanism underlying this spatially polarized protein synthesis is poorly understood but likely involves mRNA localization and localized translation since this highly conserved mechanism occurs across species and cell types to ensure positioning of nascent proteins precisely where they are needed. It is not known whether a Netrin-1 gradient causes asymmetric translation in growth cones through selectively engaging pre-localized mRNAs closest to the site of signal reception and/or whether it elicits the polarized recruitment or transport of mRNAs to that site from distant areas.

Many mRNA species exhibit axonal or growth cone enrichment relative to their levels in the soma (Zivraj et al., 2010; Gumy et al., 2011; Shigeoka et al., 2016) and different mRNAs can be specifically recruited into, or withdrawn from, the growth cone by extracellular cues such as neurotrophins, myelin-associated glycoprotein (MAG) and Sema3A (Zhang et al., 2001; Willis et al., 2007), arguing for the existence of selective mechanisms of axonal mRNA transport linked to extrinsic signals. Using fluorescent *in situ* hybridization (FISH), we have previously shown that a gradient of Netrin-1 leads to an increase in β-actin mRNA on the near-side of the growth cone within 5 min (Leung et al., 2006). This cue-induced mRNA asymmetry is consistent with a mechanism of cue-induced transport directed to the site of highest signal reception (gradient near-side) but, equally, it could arise through regulated mRNA degradation on the far-side, or selective “unmasking,” of β-actin mRNA from tightly packaged complexes that are inaccessible to *in situ* probes (Buxbaum et al., 2014). In migrating fibroblasts, β-actin mRNA localization is dependent on a 54-nucleotide “zipcode” sequence in the 3′ untranslated region (3′UTR) known to bind a regulatory RNA-binding protein, ZBP (zipcode binding protein) (Kislauskis et al., 1993, 1994; Ross et al., 1997). Consistent with the involvement of this 3′UTR sequence in cue-induced re-localization of β-actin mRNA in *Xenopus* retinal ganglion cells (RGCs), the Xenopus ZBP homolog, Vg1RBP1, also localizes to the Netrin-1 gradient near-side of growth cones (Leung et al., 2006). However, these localization studies were done at single time points in fixed samples and it is not clear whether the ribonucleoprotein particle (RNP) behavior brings about the changes in mRNA distribution (Bullock, 2011; Doyle and Kiebler, 2011) or whether there is cue-polarized transport.

To visualize directly the dynamics of β-actin mRNA in response to extrinsic signals, we used spinning-disc microscopy to capture time-lapse sequences of fluorescent-tagged mRNA in live RGC axons and growth cones. In the axon, we demonstrate that the mRNA undergoes bidirectional, microtubule-dependent movement and that global Netrin-1 stimulation rapidly enhances anterograde mRNA transport. In the growth cone, we show that a global Netrin-1 stimulation promotes the re-localization of β*-actin* mRNA from the central domain to the periphery. An attractive Netrin-1 gradient elicited a rapid, short-lived polarization of β*-actin* mRNA to the near-side of the growth cone, transducing the polarity of the extrinsic guidance cue. The β*-actin* 3′UTR was required for these cue-induced local mRNA re-localization events. Netrin-1-induced excursions of mRNA into the growth cone periphery were found to depend on microtubules and dual imaging revealed β-actin mRNA granules moving into filopodia along dynamic microtubules, behind EB1 comets. Finally, labeling endogenous RNAs with Cy-3-UTP uncovered the existence of hitherto unreported fast-moving elongated RNA structures (“worms”) that may represent a novel type of RNA-carrying organelle.

## Materials and methods

### Embryos

*Xenopus laevis* embryos, obtained by *in vitro* fertilization, were raised in 0.1 × modified Barth's saline (MBS; 8.8 mM NaCl, 0.1 mM KCl, 0.24 mM NaHCO_3_, 0.1 mM HEPES, 82 μM MgSO_4_, 33 μM Ca(NO_3_)_2_, 41 μM CaCl_2_) at 14–22°C and staged according to Nieuwkoop and Faber (Nieuwkoop and Faber, 1967). This research was regulated under the Animals (Scientific Procedures) Act 1986 Amendment Regulations 2012 following approval by the University of Cambridge Animal Welfare and Ethical Review Body (AWERB).

### Reagents

Purified Netrin-1 (R&D Systems) was used at 300 ng/ml for bath application and 10 μg/ml in a micropipette for gradient application (de la Torre et al., 1997; Campbell and Holt, 2001; Konopacki et al., 2016). Nocodazole (0.67 μM, Sigma), colchicine (0.1 μM, Sigma), and cytochalasin D (0.1 μM, Sigma) were bath-applied for cytoskeleton disruption. MitoTracker Green FM (Life Techonologies) was used at 50 nM with 30 min incubation at room temperature for live imaging of mitochondria.

### Plasmids

The pCS2-β-actin construct was generated by cloning *X. laevis* β-actin (Accession No. BC041203) with the full-length 3′UTR sequence into the pCS2+ vector. The pCS2 β actin-Δ 3′UTR plasmid contains the coding sequence of *X. laevis* β-actin and lacks the first 419 bp of the 3′UTR including the zipcode. pCS2-β-actin-Δ3′UTR was produced as follows: a plasmid was previously generated in the lab (Leung et al., 2006) in which CoralHue^TM^ Kaede was fused with *X. laevis* β-actin lacking the first 419 bp of the 461 bp 3′UTR; the Kaede sequence was removed (XhoI/BamHI) and replaced with the coding sequence of *X. laevis* β-actin, which was amplified from the pCS2-β-actin plasmid with the following primers: 5′CGCGGATCCATGGAAGACGATATTGCC3′, 5′CCGCTCGAGTTAGAAGCATTTACGGTGGAC3′. The pCS2-EB1-GFP construct consisted of DNA encoding mouse EB1 with C-terminal fusion of EGFP in a pCS2+ vector.

### RNA synthesis

Fluorescent mRNAs for live imaging were synthesized as described below. The plasmids pCS2-β actin and pCS2-β-actin-Δ3′UTR were linearized with XbaI and BssHII, respectively, and used as templates for *in vitro* synthesis of β-actin mRNA with full-length and truncated 3′UTR respectively. *In vitro* transcription was carried out using SP6 polymerase (Roche) in the presence of 0.264 mM m^7^G(5')ppp(5')G Cap analog (Ambion), 0.4 mM ATP, 0.4 mM CTP, 0.12 mM GTP, and 0.4 mM UTP. One quarter of the total UTP (0.1 mM) was conjugated with the fluorophore cyanine 3 (Cy3) (PerkinElmer), Cy5 (PerkinElmer) or Alexa 488 (Invitrogen) to render the synthetic mRNA fluorescent. Transcription reactions were incubated for 2 h at 37°C. The synthesized RNA was treated with DNAse I (Stratagene), cleaned using the RNeasy Mini Kit (Qiagen), precipitated with lithium chloride (from the mMessage mMachine Kit, Ambion), and resuspended in RNAse-free water.

EB1-GFP mRNA for expression in Xenopus RGCs was synthesized as described below. The plasmid pCS2-EB1-GFP was linearized with NotI-HF and used as template for *in vitro* transcription, which was conducted using SP6 RNA polymerase (Ambion, mMachine mMessage SP6 Kit) at 37°C for 2 hr. The products were DNase-treated, cleaned and lithium chloride-precipitated as described above.

### Electroporation

Synthetic mRNAs were introduced into Xenopus eye primordia by electroporation as described (Falk et al., 2007; Wong et al., 2017; Wong and Holt, 2018). Briefly, stage 24-27 Xenopus embryos were removed from jelly coat and anesthetized with 0.04% 3-aminobenzoic acid ethyl ester methanesulphonate salt (MS222, Sigma) in 1xMBS. The embryo was placed ventral-side-up in the longitudinal channel of a cross-shaped Sylgard chamber. The eye primordia were juxtaposed to the opening of the transverse channel, aligned with a pair of platinum electrodes positioned at the ends of the transverse channel. Synthetic mRNAs was back-loaded into a pulled borosilicate glass capillary (1 mm OD, 0.78 mm ID), and injected into the eye facing the positive electrode using an air-pressured injector. The capillary was withdrawn before a series of eight electrical pulses (18 V, 50 ms pulse duration) was delivered at one pulse per second by a square wave pulse generator. The electroporated embryos were returned to 0.1xMBS. After 2–3 h the electroporated eyes were dissected and cultured for live imaging.

For a single species of fluorescent mRNA, typically the products from a 50-μl transcription reaction resuspended in 1.5–2 μl water was in the range of 2–4 μg/μl and sufficient to electroporate 12–20 embryos. For co-electroporation of two species of fluorescent mRNAs, the concentration of each species was maintained above 1 μg/ μl in the mixture. For simultaneous live imaging of β*-actin* mRNA and GFP-tagged EB1, fluorescent synthetic mRNA (>1 μg/μl) was co-electroporated with *EB1-GFP* mRNA (0.10–0.12 μg/μl).

### Live cell imaging

Stage 24–27 retinal primordia electroporated with synthetic mRNA were cultured overnight in 60% L15 medium on coverglass coated with poly-L-lysine (10 μg/ml, Sigma) and fibronectin (1 μg/ml, Sigma). Time-lapse imaging was performed using an Olympus IX81 confocal microscope equipped with a spinning disc Yokogawa scan head (PerkinElmer) driven by the Volocity software (PerkinElmer). Time-lapse imaging was carried out using a 60X water (1.20 NA) or an Olympus 100X oil (1.40 NA) objective lens at frame rates ranging from 0.38 to 1 frame per second. Multi-channel images were acquired by a single camera using the Emission Discrimination mode of Volocity. Alexa 488, Cy3, and Cy5 fluorophores were excited with 488, 561, and 640-nm lasers respectively.

### Quantitative analysis of RNA granule kinetics and localization

The image sequences obtained from live imaging were visualized using Volocity software (Improvision). RNA granules were classified into three groups: (1) stationary or oscillatory i.e., no movement or ≤ 0.2 μm/s over 10 s; (2) with directed movement in one run >0.5 μm in growth cones or >2.1 μm (up to 31.2 μm in this study) in axons. We calculated the net displacement of β-actin mRNA granules as the summation of displacements of all granules per unit time, with retrograde displacements being assigned negative values. For characterizing the motility of RNA granules in the basal condition and upon global Netrin-1 stimulation, kymographs were generated by drawing a line along the distal axons from the image sequences using the ImageJ software. The length and angle of the line segments in the kymograph were measured using OpenLab software. Some of the images were analyzed for object movement using the manual tracking plugin of ImageJ software. The number of RNA granules passing three gating positions along the axon shaft and the number of RNA granules localizing to the growth cone periphery were scored manually. To characterize the distribution of RNA granules in the growth cone before and after Netrin-1 gradient stimulation, the images were oriented with the axon shaft along the vertical direction (y-axis) and the width of the growth cone spanning the horizontal direction (x-axis). The micropipette was always positioned to the left side of the growth cone. The x-coordinates of all the mRNA granules in the growth cone were recorded and the average calculated (X_RNA_), to give a collective representation of the location of the β-actin mRNA granules. The x-coordinate of the growth cone centroid (X_centroid_) was determined from the phase image. The relative shift values of the mRNA granules were calculated as (X_RNA_–X_RNAatTime = 0_) for **Figure 4C**, (X_RNA_–X_centroid_)/X_centroid_^*^100% for Figures 4D,E. For side-by-side asymmetry analysis of β*-actin* mRNA and peripheral dynamic microtubules, the same coordinate scoring system as described above was used. The mean displacement of all fluorescent β-actin mRNA granules from the centroid was calculated as described above at 20-s interval. For EB1-GFP, only the x-coordinates of comets outside the central domain were recorded (X_peri-*EB*1_). Due to the sporadicity of dynamic microtubule outward movement, the number of data points available for peri-EB1 was small and variable at different time points. Thus the displacement of all individual peripheral EB1-GFP comets from centroid (X_peri-*EB*1_–X_centroid)_ was summated and plotted for every time point, at 2.5-s interval.

### Growth cone turning assay

Stage 24 *X. laevis* eye primordia were cultured for 16–24 h on cover glass coated with poly-L-lysine (10 μg/ml) and fibronectin (1 μg/ml), conditions that promote attractive turning to Netrin-1. Gradients of Netrin-1 or control medium were established as described previously (de la Torre et al., 1997; Campbell and Holt, 2001; Konopacki et al., 2016). Images were taken with a 20X objective every 5 min for 60 min.

### Immunofluorescence

For detection of growth cone cytoskeletal network, RGC cultures used for live imaging were fixed (2% paraformaldehyde/7.5% sucrose, 30 min) at the end of time-lapse acquisition, permeabilized (0.1% Triton X-100 in PBS, 3 min) and blocked (5% goat serum, 20 min). Dynamic microtubules were detected using a rat anti-tyrosinated tubulin antibody (AbCam, 1:1000) followed by a goat anti-rat IgG Alexa 488 secondary antibody (Invitrogen, 1:1000). F-actin was detected using Texas-Red (Invitrogen, 1:40) or CF 405 (Biotium, 1:20) phalloidin, which was added during the secondary antibody incubation. The samples were imaged on the spinning disc microscope with the same objective lens as used for live imaging as stated earlier.

### Super resolution microscopy

For optimal optical performance, retinal cultures for super resolution microscopy were grown on 18 x18 mm^2^ high performance glass coverslips (Zeiss, thickness 0.17 ± 0.005 mm). After FISH and immunofluorescence procedures, the coverslips were mounted with Vectashield Hardset mounting medium (Vector Labs) and stored at 4°C before imaging.

Super resolution imaging was performed using a Delta Vision OMX 3D-Structured Illumination Microscope (SIM) System V3 (Applied Precision). Samples were first viewed on an Olympus IX 70 epi-fluorescence microscope to identify regions of interest (ROIs), the stage positions of which were saved. The samples were then transferred onto the OMX microscope stage, which was aligned with the IX 70 stage, and the stored regions of interest (ROIs) could be visited. Images were acquired with a 100X 1.4 NA oil objective (Olympus) with immersion oil 1.514 (ZEISS). Green and red channels were excited with 488 and 593 nm lasers respectively and captured by two separate cameras. Z-stack images were acquired at 0.125 μm steps. The raw images from the two channels were aligned in the XY dimension and deconvolved for three-dimensional reconstruction using the SOFTWORX v.5.0.0 software (Applied Precision). The aligned and deconvolved image stacks were subsequently viewed and adjusted for display using the Volocity software.

To perform unbiased quantification of the colocalization between β-actin mRNA and tyrosinated tubulin, we first performed image deconvolution and used the Volocity quantitation package (PerkinElmer) to automatically identify the mRNA puncta in 3D. We then computed a stringent “background signal” threshold by summing the fluorescence mean + 10 times the standard deviation in the tyrosinated tubulin channel from a 50 × 50 pixel in the z-stack (volumetric) next to the growth cone. Colocalization is defined by mRNA volume with mean signal of tyrosinated tubulin fluorescence that surpasses the stringent “background signal” threshold.

### *In vivo* imaging

Embryos were lightly anesthetized with 0.4 mg/ml MS222 in 1xMBS. The lateral surface of the brain contralateral to the electroporated eye was exposed by carefully removing the overlying epidermis and the contralateral eye. Embryos were mounted in an oxygenated chamber created with Permanox slides (Sigma-Aldrich) and Gene Frame (ThermoFisher), and bathed in 1xMBS with 0.1 mg/ml MS222, for visualization with fluorescence microscopy (Roque et al., 2016; Wong et al., 2017). Imaging was performed using the 60X UPLSAPO objective (NA 1.3) with a PerkinElmer Spinning Disk UltraVIEW ERS, Olympus IX81 inverted spinning disk confocal microscope. Z-stack intervals of 1.5 μm were employed for acquiring images with Volocity (PerkinElmer).

### Statistics

Data were analyzed in PRISM 7 (GraphPad). Data are presented as mean and error bars represent standard error of the mean (SEM). “n” represents the number of axons or growth cones per experiment, each of which typically included the analysis of 50-150 RNA granules per 1-s timepoint over a 5 min period. ^*^p < 0.05, ^**^p < 0.01, ^***^p < 0.001, ###*p* < 0.001. Details of statistics results are presented in the figure legends.

## Results

### Fluorescent UTP-labeled RNA movements in live axons and growth cones

**Figure 1:**
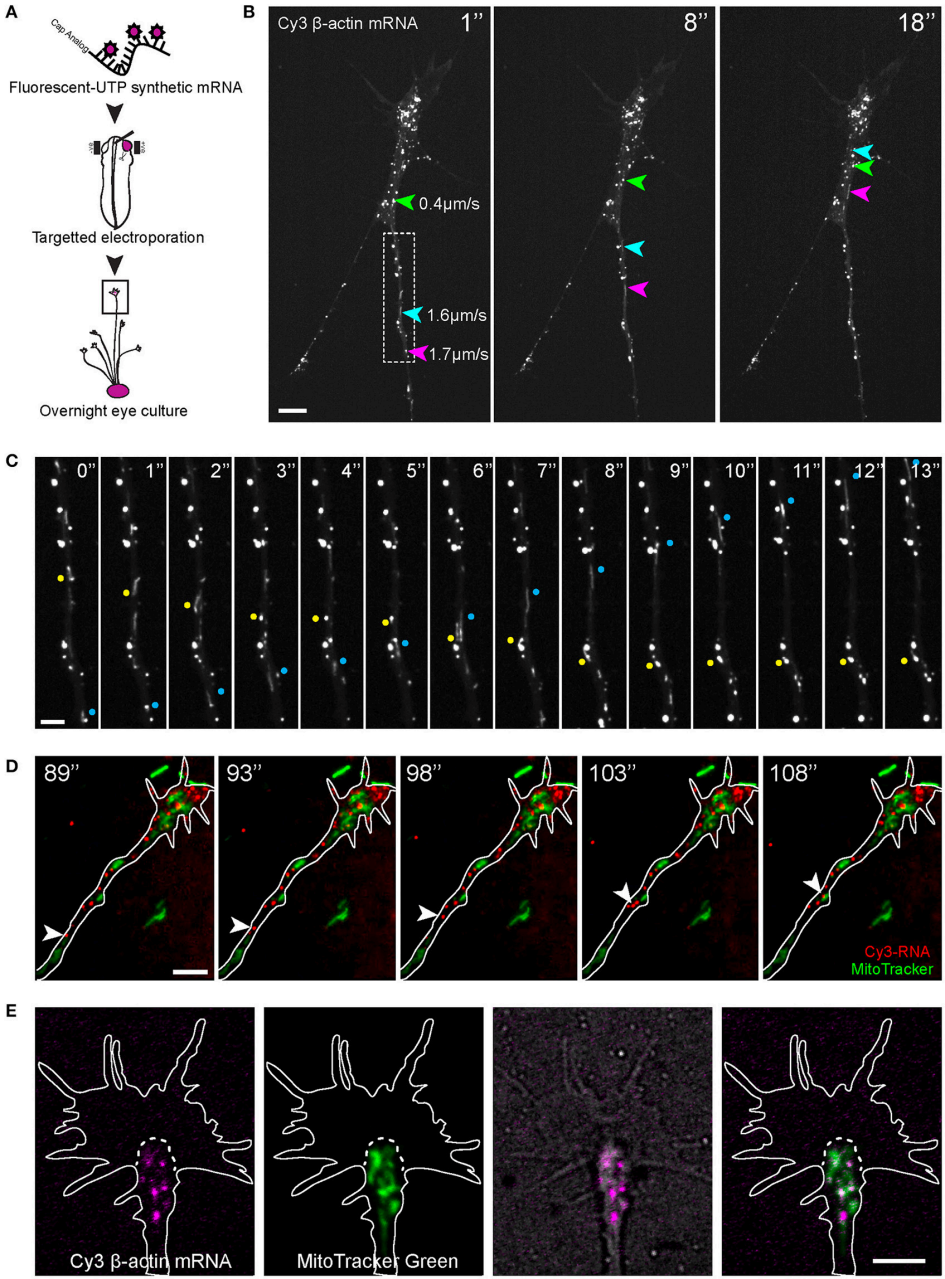
Visualization of β-actin mRNA trafficking in axons. **(A)** Fluorescent β-actin mRNA was electroporated into stage 24–27 Xenopus embryo eye primordia. The electroporated eye primordia were dissected and cultured for live imaging. **(B,C)** The synthetic β-actin mRNA was observed as fluorescent granules in cultured RGC axons and growth cones. **(B)** Live imaging of β-actin mRNA granules revealed different modes of motion and a broad range of speed (arrows and arrowhead). **(C)** Both anterograde (blue dots) and retrograde (yellow dots) movements were present. **(D)** OMX imaging of endogenous RNA labeled globally with Cy3-UTP (Cy3-RNA) and mitochondria. **(E)** Within the growth cone the β-actin mRNA granules tended to concentrate in the organelle-rich central domain, as indicated by MitoTracker Green. Scale bars, 5 μm for **(B,D,E)** and 2 μm for **(C)**. See also Figure S1.

To investigate how extracellular stimuli affect the trafficking of identified mRNAs in growing axons, we used the approach of introducing fluorescent-labeled mRNAs into retinal ganglion cell (RGC) neurons. Specific mRNAs were *in vitro* transcribed in the presence of Cy3-UTP or Alexa-488-UTP (Figure 1A) (Bullock and Ish-Horowicz, 2001; Cha et al., 2001; Wilkie and Davis, 2001) and delivered into retinal ganglion cell (RGC) precursors by electroporation into stage 23–24 embryonic eyes *in vivo* (Figure 1A). Of note, we found that Cy3-labeled mRNA encoding green fluorescent protein (GFP) gave rise to a GFP signal in embryos and in cultured RGC growth cones (Figure S1), indicating that synthetic mRNA can be recognized by the translation machinery and translated into protein.

Eye primordia electroporated with fluorescent-labeled β-actin mRNA were explanted into culture and, after axon outgrowth (12–36 h), fluorescence images were captured in axons every second, which allowed mRNA behavior to be studied in detail while avoiding substantial photobleaching. We observed bright fluorescent motile puncta in both axons and growth cones (Figures 1B,C,E, Movie S1). Such a punctate appearance is typical of mRNAs visualized in neurites (Bassell et al., 1994, 1998; Knowles et al., 1996; Lionnet et al., 2011), and presumably represents messenger ribonucleoprotein particles (mRNPs). In the rest of this study we refer to these fluorescent puncta as “mRNA granules.” In the axon shaft, β-actin mRNA granules were observed in a variety of motile states with granules moving in both anterograde and retrograde directions and at speeds that varied between 0 and 2.6 μm/s, while some granule remained stationary (Figures 1B,C). On reaching the growth cone, mRNA granules slowed down and accumulated mainly within the organelle-rich central domain (Figure 1E). Granules here often became stationary, as if anchored, but some exhibited intermittent “short runs” while others exited the growth cone and re-entered the axon to travel retrogradely toward the cell body (Movie S1).

To observe *endogenous* RNA movements, Cy3-UTP was delivered into Xenopus embryos and eyes (Piper et al., 2015; Wong et al., 2017) and visualized along with a mitochondrial marker (Figure 1D, Movie S2). Cy-UTP is incorporated broadly into RNAs synthesized in the embryo, including mRNA and ribosomal RNAs (Wong et al., 2017). Endogenous Cy3-RNA granules appeared to exhibit similar anterograde/retrograde gross dynamics as the synthetic β-actin mRNA and were observed to accumulate in the central domain of the growth cone, amongst, and distal to the mitochondria (Figure 1).

### Net mRNA transport dynamics in the axon and the cytoskeleton

**Figure 2:**
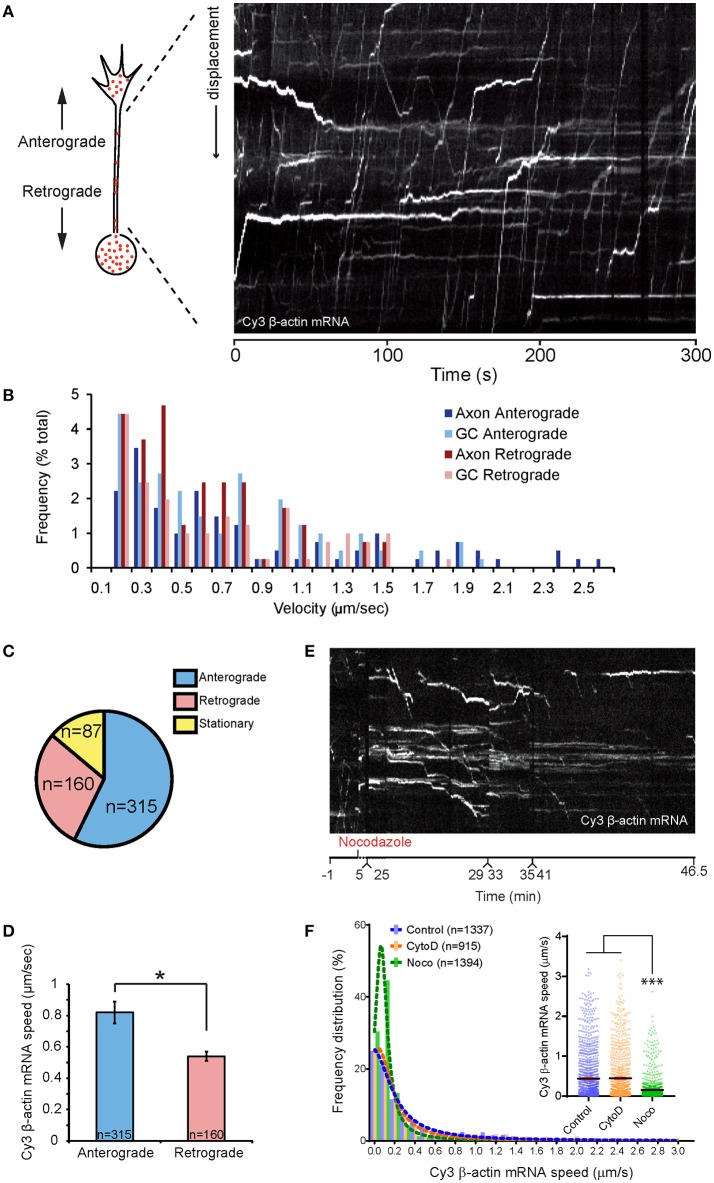
Dynamics of β-actin mRNA granules in axons. **(A)** An example of kymographs generated along axon shaft for kinetics analysis. **(B)** Population distribution of β-actin mRNA granule speeds for anterograde- and retrograde-moving granules in axon and growth cone. β-actin mRNA granule speeds showed biphasic distribution. One peak consisted of stationary and slow diffusive motion with speed < 0.5 μm/s. The more motile population showed a bell-shaped distribution. High speed (>2 μm/s) was observed for anterograde-moving granules along the axon shaft. **(C)** Proportion of anterograde-moving, retrograde-moving, and stationary β-actin mRNA granules in axons (*n* = 562 granules). **(D)** Anterograde-moving granules showed higher average speed than retrograde-moving granules. Nocodazole treatment greatly reduced kymographic tracks of fast-moving mRNA granules within 30 min, leaving mainly horizontal tracks from stationary mRNA granules. **(F)** Average instantaneous speed of all β-actin mRNA granules obtained from automated tracking is presented. The speed reduced to below 0.15 μm/sec upon 35-min nocodazole (Noco) treatment, but was unchanged upon 20-min cytochalasin D (CytoD) treatment. [*F*_(2, 3643)_ = 146, *p* < 0.0001]. Error bars represent SEM. ^*^p < 0.05, ^***^p < 0.001 (one-way ANOVA with Tukey multiple comparisons test for F).

To characterize the axonal movements of granules containing β-actin mRNA, we tracked the displacements of granules from frame-to-frame in time-lapse movies. We found that ~56% of the granules moved more than 2.1 μm (shortest run distance detectable in 7–10 min movies) in the anterograde direction (positive sloped lines in the kymograph, Figure 2A), ~30% moved retrogradely (negative sloping lines Figure 2A) and the remaining 14% were stationary (horizontal lines Figure 2A; speed ≤ 0.2 μm/s). Granules moved in the anterograde direction at an average speed of 0.8 μm/s with a maximum of 2.6 μm/s (Figures 2B–D). Movement in the retrograde direction was slower with mean and maximum speeds of 0.5 and 1.6 μm/s, respectively (Figures 2B–D). These data suggest a bias in favor of transport of β-actin mRNA in the anterograde direction, i.e., toward the growth cone.

To investigate the role of the cytoskeleton in β-actin RNA transport along the axon shaft, we applied pharmacological inhibitors of actin and microtubule dynamics. The microtubule depolymerizing agent nocodazole (0.67 μM) was added to cultured retinal axons containing fluorescently labeled mRNA granules for 35 min. A 5 min treatment with 0.1 μM nocodazole is sufficient to completely disrupt microtubules in Xenopus retinal growth cones (Piper et al., 2015). Kymographs generated from time-lapse movies showed that the number of motile granules was dramatically reduced upon nocodazole treatment, with the majority of the granules exhibiting no motion by 30 min (Figure 2E; Movie S3). The distribution of the instantaneous speed of all granules revealed a large increase in the proportion of granules with speeds below 0.2 μm/s (Figure 2F). Consequently, the average instantaneous speed of all granules was reduced from 0.45 μm/s before treatment to 0.15 μm (Figure 2F). Axons treated with the F-actin depolymerizing drug, Cytochalasin D (0.1 μM) for 20 min, however, showed speed distributions similar to those of untreated axons (Figure 2F). This concentration of Cytochalasin D disrupts the F-actin cytoskeleton within 5 min without affecting the microtubules in growth cones (Piper et al., 2015). These results indicate that the movement of β-actin mRNA granules along the axon shaft depend on microtubules rather than F-actin.

### Netrin-1 regulates β-actin mRNA transport in axons and growth cones via 3′UTR

**Figure 3:**
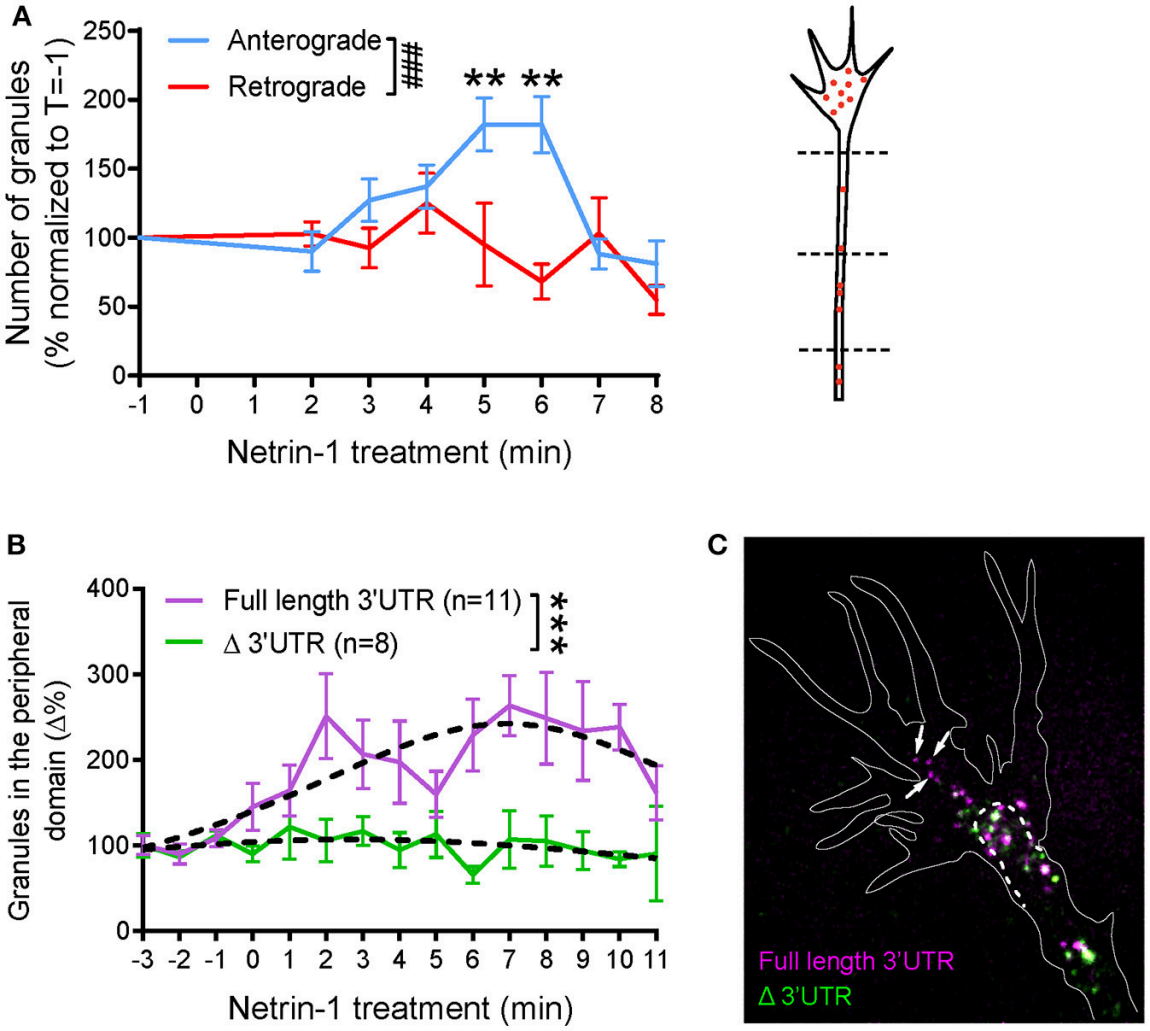
Global netrin-1 stimulation changes the dynamics of β-actin mRNA granules. Axons were imaged at 1 frame per second from 1 min before bath application of netrin-1, and the imaging continued for up to 10 min after netrin application. **(A)** The numbers of β-actin mRNA granules passing three chosen locations (dashed lines) along the axon shaft were scored. The number of anterograde-moving granules increased upon netrin-1 treatment, reaching peak value at 5–6 min and returning to the base line by 8 min of netrin-1 treatment. The number of retrograde-moving granules showed moderate decrease with time upon netrin-1 treatment. (*n* = 9 axons) Anterograde vs. retrograde [*F*_(7, 120)_ = 3.775, ^*###*^*p* = 0.001]; Vs. T = −1 min, ^**^p < 0.01 (two-way ANOVA with Dunnett multiple-comparison test) (B–C) Global netrin-1 treatment enhanced β-actin mRNA localization to the growth cone periphery in a 3′UTR-dependent manner. **(B)** Quantification of mRNA granules localizing to the growth cone periphery. Netrin-1 treatment induced a marked increase in the number of full-length β-actin mRNA granules localizing to the growth cone periphery (*n* = 11 growth cones), a response that was abrogated for β-actin mRNA with truncated 3′UTR (*n* = 8 growth cones). Dotted lines represent least-squares fits to a Lorentzian function. [*F*_(3, 178)_ = 22.01, ^***^p < 0.0001; extra sum-of-squares F test]. **(C)** Growth cones containing both full-length and Δ3′UTR β-actin mRNAs were fixed after 5-min bath netrin-1 treatment. The full-length mRNA granules localized further into the growth cone periphery (white arrows) while the Δ3′UTR granules remained in the central domain. Error bars represent SEM. See also Figure S2.

To investigate whether Netrin-1 alters axonal β-actin mRNA transport, we imaged axons 1–2 min before, and at least 5 min after, Netrin-1 was applied globally to the culture medium. In the axon shaft, the average velocity of mRNA granules showed an upward trend 2–6 min post-Netrin addition (Figures S2A,B), but this was not statistically significant. Both anterograde and retrograde granules exhibited similar velocities (Figure S2B). The displacement per track, also known as “run length,” increased significantly 2-6 min post-Netrin incubation in both anterograde and retrograde directions (Figures S2A,C). As a further measurement to examine directional bias in β-actin mRNA granule flux, we used the stringent criterion of counting the number of granules that crossed all three “gates,” spaced ~10 μm apart, along the axon (see diagram Figure 3A). We then recorded their anterograde and retrograde trafficking directions along the axon before and up to 8 min after Netrin-1 addition (Figure 3A). These results revealed a clear and transient increase in anterograde transport of β-actin mRNA, peaking 4–6 min after Netrin-1 addition, and returning to baseline levels by 8 min. No changes were detected in the retrograde direction within this timeframe. Together the results show that the increased proportion of anterograde granules results in a net flux of β-actin mRNA into the distal axon in response to Netrin-1. Changes in granule velocity and displacement do not appear to underlie this cue-induced anterograde bias (Figure S2), however, suggesting that changes in other factors, such as motor protein usage or affinities to different RNA binding proteins (RBPs), may be involved.

We next investigated if global addition of Netrin-1 changes the distribution of β-actin mRNA granules within the growth cone. Cy3 β-actin mRNA containing either the full length (FL) 3′UTR or truncated (Δ)3′UTR were introduced separately into the cells for visualization. Before Netrin-1 treatment, most of the β-actin mRNA granules with FL 3′UTR clustered within the organelle-rich phase-dense “central” domain of the growth cone with only occasional transport of granules from the central domain to the peripheral area in basal conditions. After addition of Netrin-1, a gradual increase in the number of peripheral granules was observed up to at least 8 min after Netrin-1 addition (Figure 3B; Movie S4), by which time point, there was a 2-3-fold increase in the number of β-actin mRNA granules in the periphery. This peripheral-directed transport of β-actin mRNA granules was completely dependent on the 3′UTR because β-actin mRNA lacking the first 419 nt of its 3'UTR, including the zipcode sequence (β-actin-Δ3′UTR), did not relocalize to the growth cone periphery on Netrin-1 stimulation (Figure 3B). The increased movement of granules into more peripheral parts of the growth cone is similar to that seen with Vg1RBP-GFP with global Netrin-1 stimulation, and with cell contact (Leung et al., 2006), and suggests coordinated coupling between extrinsic signals and RNP transport. These observations are consistent with Netrin-1-induced translation enhancement in the peripheral region of growth cone, as visualized by single molecule translation imaging (Ströhl et al., 2017), and with contact-induced peripheral trafficking of Vg1RBP (Leung et al., 2006).

To investigate further the 3′UTR-dependent cue-induced mRNA transport differences, we labeled growth cones simultaneously with both β-actin mRNA species (with FL and Δ 3′UTR) using different colored fluorescent tags. The differential trafficking pattern observed from separate expression of the two β-actin mRNAs (Figure 3B) was confirmed by co-imaging β-actin FL 3′UTR and Δ3′UTR mRNA species in the same growth cone (Figure 3C). After 5 min of bath Netrin-1 treatment, FL 3′UTR mRNA granules were seen to localize further toward the growth cone periphery than the Δ3′UTR*-* β-actin mRNA granules (Figure 3C).

### Netrin-1 gradient induces polarized mRNA transport in growth cones dependent on 3′UTR

**Figure 4:**
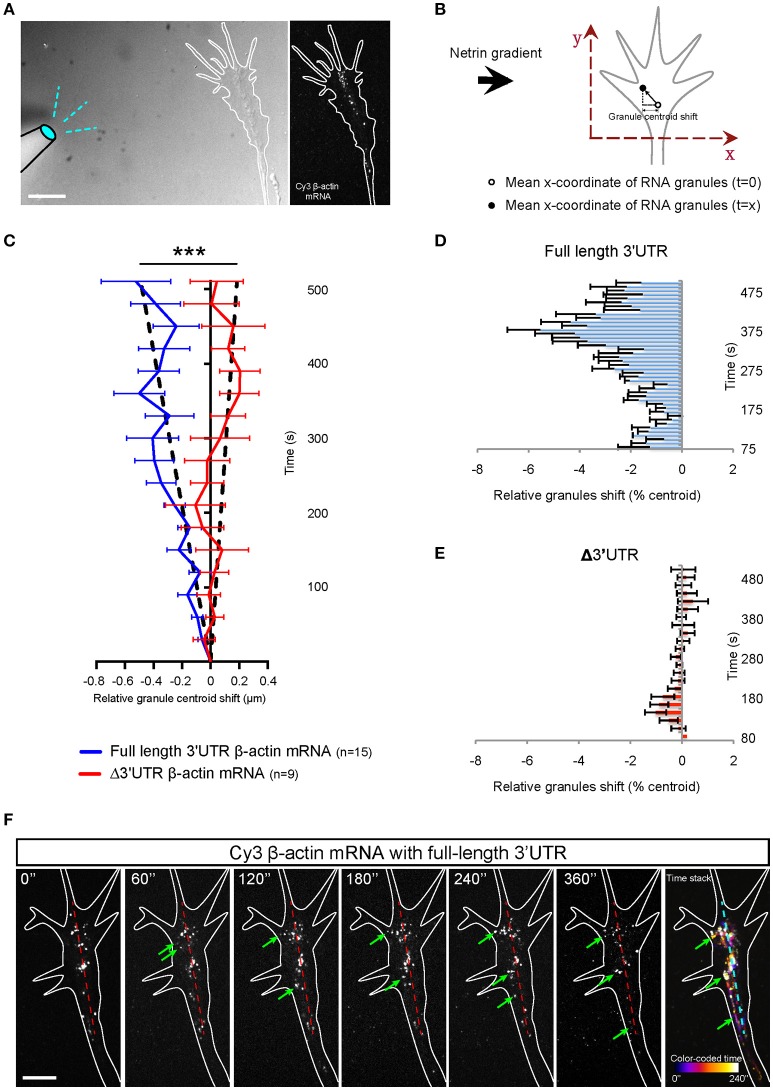
Netrin-1 gradient induces asymmetric localization of β-actin mRNA in growth cone. **(A)** Growth cones were imaged at 1 frame per second in the presence a netrin-1 gradient set up from a micropipette perpendicular to the growth cone. **(B)** The mean x-coordinate of all β-actin mRNA granules at each timepoint was compared to the mean x-coordinate at time 0. With the source of netrin-1 on the left, a negative shift value indicates a shift of β -actin mRNA granules toward the netrin-1 source. **(C)** Relative granule centroid shift values are plotted. (*n* = 15 growth cones for full length 3′UTR at 1fps for 5–15 min; *n* = 9 growth cones for Δ3′UTR at 1fps for 5–15 min) Black dotted lines represent least-square fits to a linear function. [*F*_(1, 407)_ = 98.27, ^***^p < 0.0001; extra sum-of-squares F test] **(D)** The distribution of full-length β-actin mRNA granules are binned and show a rapid shift toward the source of netrin-1. The bar chart shows the averages from 3 growth cones. **(E)** β-actin mRNA with truncated 3′UTR did not show asymmetric distribution toward netrin-1 gradient. The bar chart shows the averages from 6 growth cones. **(F)** Time lapse images showing a prominent shift of full-length β-actin mRNA toward the source of netrin gradient (toward the left as denoted by green arrows; midline is denoted by dashed red line. (**F**; far right) Photobleaching-corrected, denoised time lapses images were stacked and maximally projected. The temporal-color coded image reveals the full-length β-actin mRNA shift toward netrin gradient is both asymmetrical and fast. Scale bars, 5 μm. Error bars represent SEM.

A gradient of Netrin-1 induces asymmetrical β-actin synthesis in RGC growth cones on the near-side which may be aided by the polarized transport of Vg1RBP carrying β-actin mRNA (Leung et al., 2006). To chart the dynamics of β-actin mRNA during gradient presentation, we followed Cy3-labeled β-actin mRNA, with and without the 3'UTR, in growth cones at 1 frame per second for ~10 min following the onset of a Netrin-1 gradient presented at a 90° angle (a time window just before growth cone steering or centroid shift). For each time point for each growth cone, we calculated the mean x coordinate value of the mRNA granules relative to time 0. (Figures 4A,B). Growth cones showed a shift in the distribution of β-actin mRNA with the full length 3′UTR toward the gradient, starting around 60 s (Figures 4C,D,F). The average values show that within 5 min of exposure to a Netrin-1 gradient, the collective distribution of β-actin mRNA granules with the full length 3′UTR shifted toward the source of Netrin-1 (Figures 4C,D,F). By contrast, no shift was evident with the mutant Δ3′UTR β-actin mRNA (Figures 4C,E). We compensated for the variability associated with positioning the pipette (time of gradient onset), by aligning the individual curves by the time point of greatest asymmetry. This gave a pronounced V-shaped profile with the full length 3′UTR β-actin mRNA (Figures 4C,D) indicating that the asymmetric shift toward the source of Netrin-1 reaches a maximum 4–6 min after gradient onset, and returns toward symmetry by ~8 min.

Together, these observations suggest that β-actin mRNA trafficking can be specifically regulated by the guidance cue Netrin-1 and that such regulation requires the 3'UTR of β-actin, containing the zipcode sequence.

### Microtubules are required for β-actin mRNA transport into the growth cone periphery

**Figure 5:**
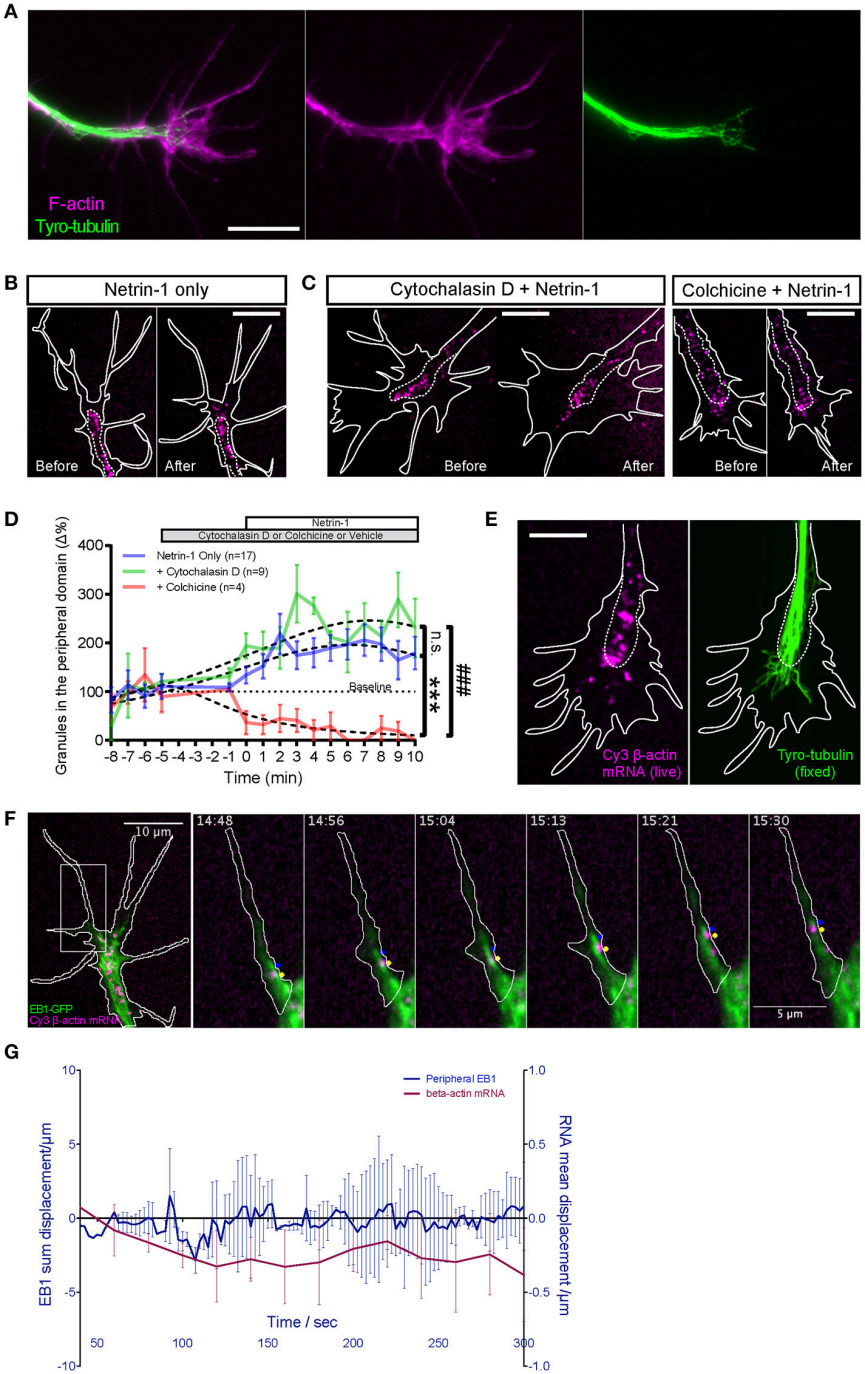
Netrin-1 induced RNA movement to growth cone periphery depends on dynamic microtubules and not F-actin. **(A)** Fluorescence micrographs show the relative distributions of F-actin (phalloidin staining) and dynamic microtubules (tyryosinated-tubulin) in distal axon. **(B–C)** Dynamic microtubule and F-actin were selectively disrupted to test which cytoskeletal element was required for facilitating netrin-1 induced peripheral mRNA localization. Unlike with intact cytoskeleton (**B**, Netrin-1 only; *n* = 17 growth cones), disrupting dynamic microtubules with colchicine (**C**, Colchicine + Netrin-1; *n* = 4 growth cones) abolished the netrin-1-induced increase in peripheral mRNA localization. In the presence of cytochalasin D (**C**, Cytochalasin D + Netrin-1; *n* = 9 growth cones), which disrupts F-actin, mRNA granules still exhibited increased peripheral localization in response to netrin-1 stimulation. Each panel in **(B,C)** shows the same growth cone before and after netrin-1 treatment. **(D)** Quantification of the number of peripherally localized mRNA granules. Dotted lines represent least-squares fits to a Lorentzian function. (Netrin vs. Netrin + Cytochalasin D: *F*_(3, 243)_ = 2.405, *p* = 0.068; Netrin vs. Netrin + Colchicine: *F*_(3, 222)_ = 25.75, ^***^p < 0.0001; Netrin + Cytochalasin D vs. Netrin + Colchicine: *F*_(3, 119)_ = 57.09, ###*p* < 0.0001; extra sum-of-squares F test) **(E)** The peripheral distribution of Cy3- β-actin mRNA granules in live, netrin-1 bath treated growth cones (left) was compared to that of dynamic microtubules (right), which was revealed by tyrosinated tubulin immunostaining after immediate fixation of the live growth cones. The distance by which dynamic microtubules probed into growth cone periphery was comparable to that reached by netrin-1-recruited peripheral mRNA granules. **(F)** Live imaging of Cy3-β-actin mRNA granules with GFP-tagged EB1 captured outward-moving mRNA granules in filopodia trailing behind EB1-GFP comets that tipped the dynamic microtubules (image sequence of region in square). **(G)** Dynamic microtubules in growth cone periphery do not show asymmetric distribution concurrent with or prior to β-actin mRNA polarization upon netrin-1 gradient stimulation. (*n* = 4 growth cones) Scale bars, 10 μm. Error bars represent SEM.

Our results showing that microtubules, not actin, are necessary for β-actin mRNA transport along the axon shaft is not surprising since microtubules predominate in axons (Dent et al., 2011) and mRNAs have previously been shown to associate with microtubules in neurons (Bassell et al., 1998), and require intact microtubules for long-range and stimulus induced axonal transport (Zhang et al., 2001; Muslimov et al., 2002; Willis et al., 2007). In the growth cone, however, the actin cytoskeleton predominates, at least in the periphery, with stable microtubules being confined mostly to the central domain (Geraldo and Gordon-Weeks, 2009). The periphery also contains dynamic microtubules, which are constantly polymerizing and depolymerizing and these can extend far into filopodia (Lowery and Van Vactor, 2009; Dent et al., 2011) (Figure 5A). We wondered, therefore, whether the transport of β-actin mRNA into the growth cone periphery was dependent on dynamic microtubules, or whether the granules switched tracks onto actin filaments. We found that β-actin mRNA granules still exhibited increased peripheral localization following Netrin-1 in the presence of 0.1 μM Cytochalasin D, which disrupts F-actin without affecting dynamic microtubules (Figures 5B–D) (Piper et al., 2015). By contrast, a short 12.5μM colchicine treatment, which eliminates dynamic microtubules while leaving F-actin intact (Piper et al., 2015), effectively abolishes the Netrin-1 induced increase in β-actin mRNA peripheral localization (Figures 5B–D). This result indicates that β-actin mRNA granules move into the periphery on dynamic microtubules.

### β-actin mRNA travels on dynamic microtubules into the growth cone periphery

To directly test if peripherally localized mRNA granules interact with dynamic microtubules, growth cones containing Cy3 β-actin mRNA were bath-treated with Netrin-1 and monitored in real time. Upon observing increased mRNA granule outward movements, the growth cone was fixed immediately and immunostained for tyrosinated-tubulin (tyro-tubulin), a marker for dynamic microtubules (Kreis, 1987; Challacombe et al., 1997). The Cy3 fluorescence of the β-actin mRNA could not be preserved through fixation and immunostaining, so the distribution of the Cy3-β-actin mRNA granules captured live immediately before fixation was compared to that of the dynamic microtubules (following immunostaining) (Figure 5E). These non-simultaneous images show that dynamic microtubules can extend peripherally to distances comparable to those reached by Netrin-1-recruited mRNA granules, for example, to the edge of lamellipodia and the base of filopodia.

To observe the dynamic relationship between Cy3-β-actin mRNA granules and dynamic microtubules, we performed dual live imaging with Cy3-β-actin mRNA and GFP-tagged EB1, a microtubule plus-end-tracking protein (Mimori-Kiyosue et al., 2000; Morrison et al., 2002; Matov et al., 2010) (Figure 5F; Movies S5, S6). Netrin-1 was bath applied to elicit movements of mRNA granules toward the periphery. Live imaging captured multiple examples of peripheral-moving mRNA granules trailing behind EB1 comet-marked tips of dynamic microtubules (Figure 5F, Movies S5, S6). β-actin mRNA granules were never observed to overtake an EB1 comet but remained behind, often traveling together in tandem. These results provide direct visual evidence of β-actin mRNA granules traveling to the growth cone periphery on dynamic microtubules. Together, these results show that intact dynamic microtubules, but not F-actin, facilitate the Netrin-1 induced peripheral localization of β-actin mRNA granules in growth cones and suggest that this is achieved by direct association with, and outward movement on, dynamic microtubules.

Given the close dynamic relationship between β-actin mRNA granules and EB1 comets, we wondered whether the polarized transport of β-actin mRNA granules induced by a Netrin-1 gradient could be mediated by polarized dynamic microtubules. Therefore, we compared the displacement values of EB1 comets and β-actin mRNA granules in growth cones over 5 min in a Netrin-1 gradient. EB1 showed no detectable shift whereas β-actin mRNA granules were displaced toward the gradient (Figure 5G). This result indicates there is no dynamic microtubule asymmetry induced by Netrin-1 and, therefore, it is not the direct cause for mRNA polarization, suggesting that it is a selective process.

**Figure 6:**
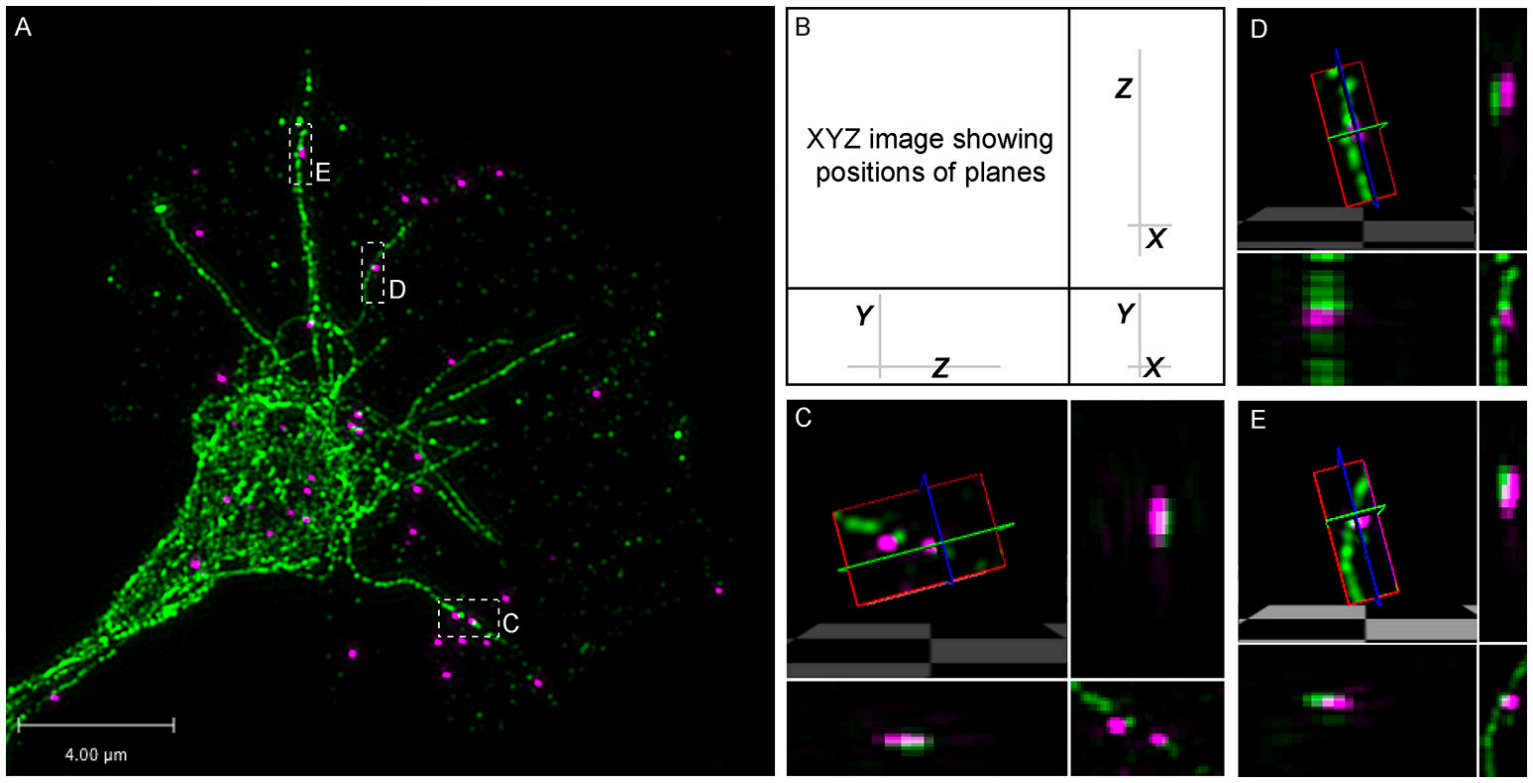
Super-resolution microscopy shows direct association between β-actin mRNA and dynamic microtubules. **(A–E)** Fluorescence *in situ* hybridization (FISH) for β-actin mRNA was followed by immunostaining for tyrosinated-tubulin in growth cone. **(B–E)** FISH puncta overlap with dynamic microtubules and 3-D rendering supports direct association. 64.08 ± 6.44% of β-actin mRNA colocalized with dynamic microtubule (*n* = 8 growth cones; 280 identified β-actin mRNA granules). Scale bar, 4 μm.

To confirm physiological relevance, we performed dual channel super-resolution imaging of *endogenous* β-actin mRNA and microtubules with Delta Vision OMX 3D-Structured Illumination Microscope (SIM) System V3. For this, fluorescence *in situ* hybridization (FISH) for β-actin mRNA was followed by immunostaining for tyrosinated tubulin in growth cone. This high-resolution dual imaging showed multiple clear examples of FISH labeled puncta in close association with a dynamic microtubule and 3-D rendering revealed this association to be direct (Figure 6). Unbiased quantitative colocalization analysis on deconvolved stacks with axial resolution of 125 nm was performed. The results reveal that 64.08 ± 6.44% of β-actin mRNA colocalized with dynamic microtubules (*n* = 8 growth cones; 280 identified β-actin mRNA granules), supporting an endogenous role of dynamic microtubules in β-actin mRNA delivery to the growth cones periphery.

### Elongated highly motile “rna worms” in axons *in vitro* and *in vivo*

**Figure 7:**
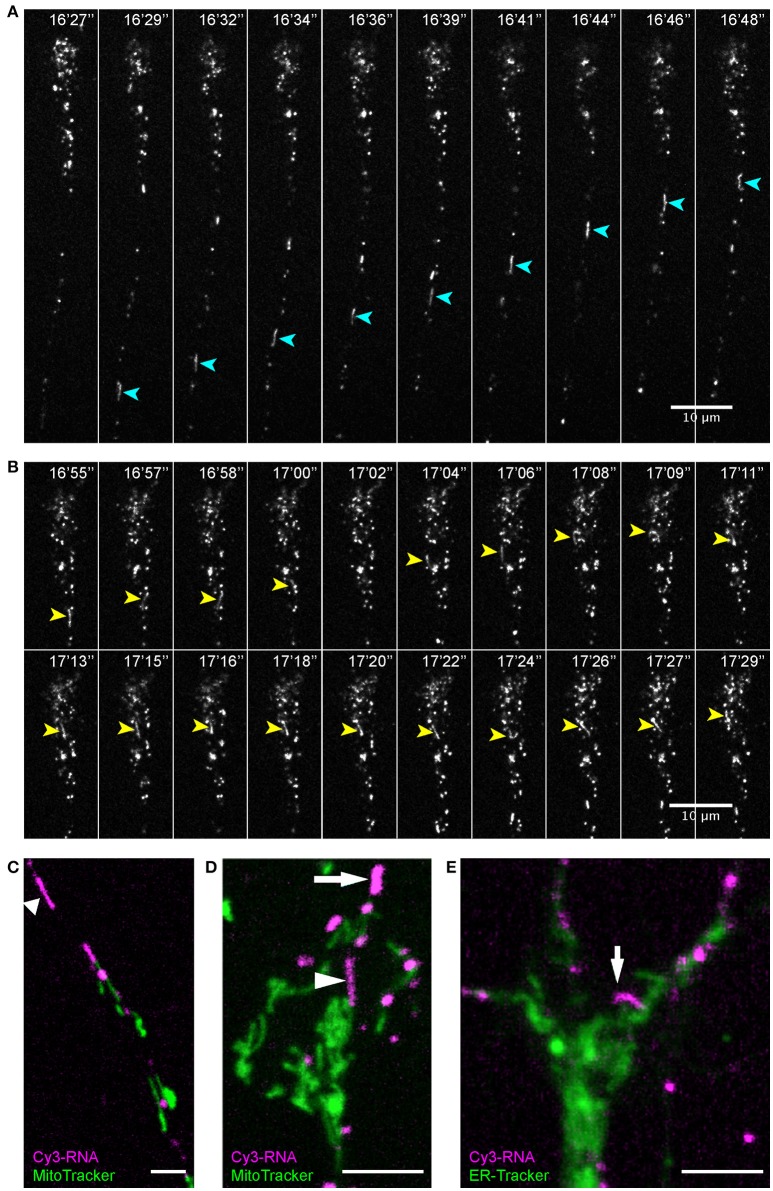
Endogenous RNA worms *in vitro*. **(A–E)** Endogenous RNA was labeled by fluorescent-UTP. Elongated RNA species, which we termed “RNA worms,” were sporadically observed during live imaging. **(A,B)** An RNA worm showing anterograde movement in the axon shaft **(A)** and more subtle twisting-turning dynamics in the distal axon/growth cone **(B)**. **(C,D)** RNA worms are not labeled by MitoTracker, as shown in both axon shaft **(C)** and growth cone **(D)**. **(E)** RNA worms are not labeled by ER-Tracker. Scale bars, 10 μm for **(A,B)** and 5 μm for **(C–E)**. See also Figures S3–5.

**Figure 8:**
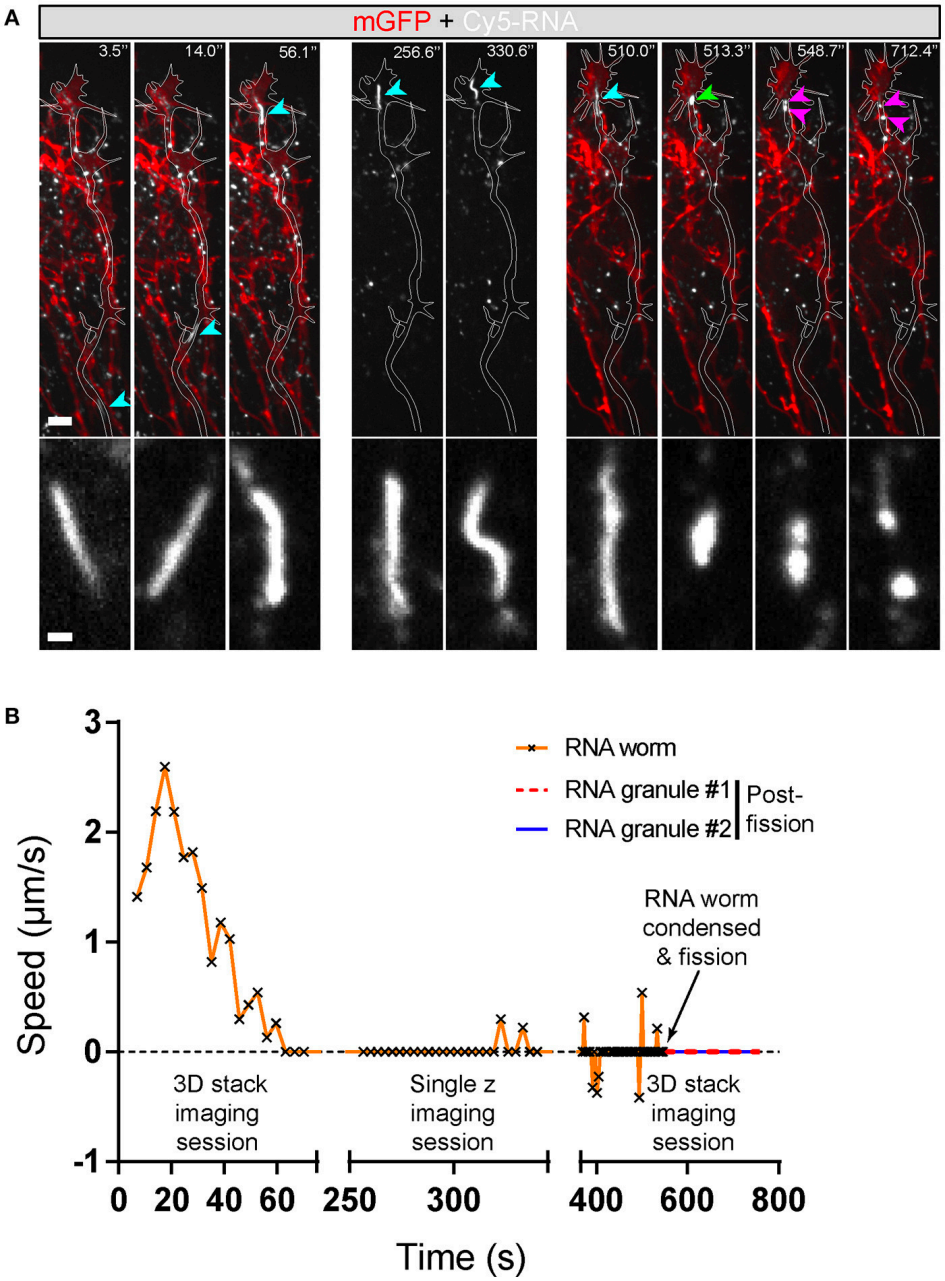
Endogenous RNA worms *in vivo*. **(A)** Endogenous RNA was labeled by fluorescent-UTP and the RGC axons were labeled by mGFP for visualization *in vivo*. The example shows the anterograde movement of the RNA worm toward the growth cone, followed by instantaneous condensation and fission into at least two separate RNA granules with globular morphologies. **(B)** Quantification of the speed of the RNA worm movement for **(A)**. Scale bars, 5 μm for upper panel and 1 μm for lower panel of **(A)**.

Live imaging of endogenous RNA in axons *in vitro* following blastomere injection of Cy3/Cy5-UTP (Piper et al., 2015; Wong et al., 2017; Cioni et al., 2018) showed that while the majority of fluorescent RNA granules exhibited a classic rounded shape, occasionally elongated worm-like motile structures were observed that, to our knowledge, have not been described previously. These striking elongated structures appeared sporadically and moved bi-directionally along axons at a rate of 1–3 μm/s and varied in length from 3–10 μm (Figure 7). They maneuvered themselves in a worm-like (flexible, non-rigid) fashion in the growth cone in quick bending and turning motions (Figure 7B; Movies S7, S8) and were often seen moving in and out of filopodia. We refer to these structures as “RNA worms.” To investigate whether they represented artifacts associated with *in vitro* culture conditions, we sought to discover whether they occurred in retinal axons *in vivo*. Live imaging of retinal axon terminals in the optic tectum also showed the existence of occasional RNA worms (Figure 8; Movie S8). Here the RNA worms can be seen moving rapidly around the terminals, twisting and turning, traveling between branches and finally splitting into several smaller round granules.

Discovering more about the identity of these structures proved challenging because the Cy3/Cy5-UTP fluorescent signal does not persist through fixation and immunostaining protocols. Therefore, we used live organelle trackers and conducted dual imaging on axons *in vitro*. First, we used MitoTracker to test whether these structures represent mitochondria since mRNAs have been described in association with mitochondria (Lesnik et al., 2015). When dual-imaged with MitoTracker, the Cy3-RNA worms did not exhibit any co-localization with MitoTracker (Figures 7D,E; Figure S3; Movies S9, S10). Time-lapse sequences showed these UTP-labeled structures to be separate entities often passing close by mitochondria, and sometimes slowing down or pausing temporarily while brushing back-and-forth against a mitochondrion (Movie S9) (Wong et al., 2017). The RNA worms were generally much more motile than mitochondria in axons and tended to move at faster speeds (1–3 vs. ≤ 1 μm/s).

Next we performed dual live imaging with ER-Tracker to test for an association with the endoplasmic reticulum. ER-Tracker showed abundant label throughout the axons and growth cone but the RNA-worms did not show obvious co-localization with the ER marker (Figure 7E; Movie S11). Similarly, there was little overlap in fluorescent labeling between Cy3-RNA granules and LysoTracker, although we were unable to capture elongated worms in these experiments. Since Cy3-UTP is incorporated into all types of RNA, including ribosomal structural RNA, it is possible that these labeled elongated structures represent RNA-ribosome-carrying organelles.

## Discussion

Here we investigated the axon's ability to alter its local mRNA distribution in response to extracellular cues during development. By looking at how β-actin mRNA moves in axons upon Netrin-1 stimulation, we found that anterograde mRNA transport is specifically and transiently increased in the axon shaft and that within the growth cone itself, β-actin mRNA moves peripherally toward the side of stimulation. Previous studies have demonstrated that particular mRNAs become enriched or depleted within axons in response to external stimuli (Willis et al., 2005, 2007; Leung et al., 2006; Yao et al., 2006; Andreassi et al., 2010). Although altered mRNA trafficking has been proposed to underlie these observed changes in RNA localization, cue-stimulated changes in mRNA transport has yet to be directly visualized, thus our understanding of the contribution of stimulus-induced mRNA trafficking has so far remained limited. Our live-imaging approach has allowed us to map the precise time-line over which altered axonal β-actin mRNA trafficking acts. We found a transient peak in anterograde transport and peripheral growth cone localization within 5 mins of Netrin-1 addition. This mRNA polarization may be sufficient to support the rapid onset of local protein synthesis, since recent single molecule translation methods have shown that newly synthesized β-actin is detectable from 1-2 min of Netrin-1 stimulation in growth cones (Ströhl et al., 2017) and proteomic analysis has identified hundreds of axonally synthesized proteins within 5 min (Cagnetta et al., 2018).

Our findings raise the question of how such a brief change in β-actin mRNA granule transport velocity and polarization, peaking on a timescale of 4–6 min can contribute to axon navigation *in vivo*, which typically takes hours to days. We speculate that the brief polarized mRNA recruitment helps to localize newly synthesized proteins to the near-side of the Netrin gradient (site of stimulus reception) and, hence, requires to be switched off after stimulus receipt as part of an adaptive mechanism to prevent “over steering” of axons. Together with other guidance cues, such as Sema3A and BDNF (Cagnetta et al., 2018), this on/off switch may provide a resettable trigger for repeated rounds of local steering throughout the journey of axon navigation. Indeed, the timescale fits well with the 5 min cycles of protein synthesis- and endocytosis-dependent re/de-sensitization that underlie growth cone cue adaptation (Piper et al., 2005). In addition, it is notable that our recent *in vivo* findings indicate that axon branching has a much higher demand for local protein synthesis than long-range axon navigation (Shigeoka et al., 2016; Wong et al., 2017; Cioni et al., 2018) where initiation of new branches is associated with mRNA docking and localized bursts of β-actin synthesis. Collectively, the findings point to a model whereby complex axon trajectories and arbors are built, step-by-step, through many separate short (e.g., 5 min) bursts of RNA-based changes (transport and translation) in response to localized extrinsic cues which eventually lead to enduring structural remodeling.

Stimulus-induced changes in axonal β-actin mRNA localization have previously been shown to be microtubule-dependent (Zhang et al., 2001). Our data support these findings showing that β-actin mRNA movement is halted upon pharmacological disruption of the microtubule cytoskeleton but not the F-actin cytoskeleton. Of particular interest is that β-actin mRNA was found to travel along dynamic microtubules even in the growth cone periphery and into filopodia. This region of the growth cone is largely comprised of F-actin filaments (Gomez and Letourneau, 2014). It was therefore surprising to find that β-actin mRNA is transported along dynamic microtubules that can extend into the periphery, particularly because short-range RNA movement in axons has previously been demonstrated to require actin (Muslimov et al., 2002). It is possible that microtubule-associated motor proteins driving β-actin mRNA movement may still cooperate with actin filament-associated motor proteins to regulate subtleties of axonal mRNA transport that we did not explore in this study. For example, anterograde microtubule driven transport of ZBP1 in axons, the Vg1RBP homolog, has been demonstrated to be negatively regulated by ZBP1's association with the actin motor: Myosin-Va (Nalavadi et al., 2012). Moreover, myosin association could also provide a sorting mechanism for axonal entry of mRNA-containing ribonucleoprotein, as has been described for proteins (Arnold, 2009). It would be interesting in future to investigate whether actin-microtubule cooperation may modulate more refined aspects of axonal β-actin mRNA localization and targeting. Nonetheless, our findings demonstrate that intact F-actin networks are dispensable for Netrin-1 induced changes in β-actin mRNA trafficking, highlighting the importance of axonal microtubule-driven transport for stimulus-induced mRNA localization.

How might Netrin-1 induce polarized changes in β-actin mRNA distribution within axons? We found that microtubule plus-end binding EB1 comets did not display asymmetry in response to a Netrin-1 gradient, demonstrating that the altered β-actin mRNA trafficking behavior we observed was not a result of polarized cytoskeletal dynamics, but instead restricted to the mRNA cargo itself. Unlike our observations for full-length fluorescently tagged β-actin mRNA, we found that β-actin mRNA lacking a 3′UTR did not exhibit peripheral growth cone localization in response to Netrin-1 stimulation. This altered trafficking behavior is likely dependent on RBPs such as Vg1RBP that binds the β-actin mRNA 3′UTR and is necessary for Netrin-1 induced β-actin mRNA enrichment in the growth cone (Leung et al., 2006; Welshhans and Bassell, 2011). Because anterograde microtubule-dependent transport is specifically affected, the 3′UTR dependent changes in mRNA trafficking we observe are likely to involve the kinesin motor protein family. Interestingly, Src kinase dependent Vg1RBP/ZBP1 phosphorylation has been shown to be triggered in response to attractive guidance cues, is required for stimulus-induced recruitment of β-actin mRNA into axonal growth cones, and is necessary for the subsequent attractive growth cone steering (Sasaki et al., 2010; Welshhans and Bassell, 2011; Lepelletier et al., 2017). Perhaps counter-intuitively, however, such phosphorylation negatively regulates ZBP1 motility and Kif5 binding in neuronal dendrites (Urbanska et al., 2017). ZBP1 has a net retrograde flow in axons (Nalavadi et al., 2012) and phosphorylation of this RBP reduces its binding to β-actin mRNA (Hüttelmaier et al., 2005). One possible explanation, therefore, is that Netrin-1 stimulates release of β-actin mRNA from Vg1RBP/ZBP1 in axons, allowing association with new/additional RBPs (Rossoll et al., 2003; Glinka et al., 2010; Fallini et al., 2014) and motor proteins that promote β-actin mRNA anterograde transport and peripheral growth cone localization. Alternatively, reduced motility via Vg1RBP phosphorylation might serve as an anchoring mechanism after β-actin mRNA reaches the growth cone periphery.

The observation that β-actin mRNA lacking a 3′UTR is transported into axons presents the interesting possibility that the 3′UTR zipcode might not be required for basal β-actin mRNA localization in axons. Indeed, knockout of the RBP ZBP1, as well as antisense oligonucleotides against the β-actin mRNA zipcode sequence results in comparable β-actin mRNA levels in axonal growth cones under basal, unstimulated conditions (Zhang et al., 2001; Yao et al., 2006; Welshhans and Bassell, 2011). Alternatively, overexpression of exogenous mRNA might lead to non-specific axonal targeting. However, since the same amount of mRNA was electroporated in both conditions, we expect any non-specificity to be the same in both conditions.

Use of fluorescent exogenous *in vitro* synthesized β-actin mRNA enabled us to directly visualize β-actin mRNA dynamics in living axons as brightly labeled puncta. While we demonstrated that exogenous fluorescently tagged mRNA could be translated, this approach also offered the unique advantage of allowing us to explore sequence specific dependencies in β-actin mRNA trafficking. Here, we found that β-actin mRNA lacking its 3′UTR did not exhibit the same trafficking dynamics as full-length β-actin mRNA in response to Netrin-1 stimulation. Previously, it has been shown that exogenous fluorescent mRNA is able to mimic endogenous mRNA localization patterns in living cells and, more recently, within *in vitro* reconstituted mRNPs (Bullock and Ish-Horowicz, 2001; Cha et al., 2001; Wilkie and Davis, 2001; Amrute-Nayak and Bullock, 2012). Our approach extends the use of this technique to neuronal axons, providing a useful tool for exploring the roles of cis-acting elements in axonal mRNA trafficking.

It is of note that mRNAs often require co- and post-transcriptional nuclear processing to achieve proper localization through interaction with specific RBPs in the nucleus (Palacios, 2002; Kress et al., 2004; Pan et al., 2007). Because delivery of exogenous mRNA into the cytoplasm bypasses such nuclear processing events and can result in over-expression, use of fluorescent *in vitro* transcribed mRNA may not entirely recapitulate localization patterns and trafficking behavior of their endogenous mRNA counterparts, as has been observed previously (Cha et al., 2001). To overcome these limitations, it may be necessary to compare exogenous mRNA dynamics to visualization of endogenous mRNA using alternative techniques such as molecular beacon technology (Tyagi and Kramer, 1996) or the MS2-GFP system (Wang et al., 1991; Bertrand et al., 1998). Indeed, while the basal dynamics we observed for Cy-UTP β-actin mRNA is remarkably similar in regards to movement type and speeds to that recently observed for single endogenous β-actin mRNA molecules in axons (Turner-Bridger et al., in press) and in dendrites (Buxbaum et al., 2014; Park et al., 2014), a key difference is that a substantially smaller fraction of exogenous β-actin mRNA remains stationary. These data suggest that while exogenous mRNA is able to mirror stimulus-induced trafficking changes in axons, nuclear processing might be required for anchoring of β-actin mRNA within the axon shaft.

By pan-labeling endogenous RNA in axons through blastomere injection of Cy5-UTP into early embryos (Piper et al., 2015; Wong et al., 2017), we were able to observe a new kind of RNA structure. These occasionally observed RNA “worms” possessed an elongated structure that was distinct from the classic punctate appearance of Cy5-UTP labeled β-actin mRNA and the majority of other Cy5-UTP labeled endogenous RNA granules. Our previous biochemical analysis has shown that fluorescent UTP injected into Xenopus embryos is exclusively incorporated into RNA (Wong et al., 2017). Moreover, we have demonstrated here through live double labeling that these RNA worm structures are not mitochondria and are apparently independent of ER and lysosomes. That RNA worms are only observed with broad Cy-UTP labeling suggests that these structures may represent accumulations of structural ribosomal RNA, or trains of ribosomes. It will be interesting in future to find out whether ribosomal RNAs or ribosomal proteins co-localize with RNA worms, or whether they are independently transported. Their sporadic frequency and the fact that the fluorescence signal of Cy-UTP does not survive through post-fixation processing make these challenging structures to work with. However, it is tempting to speculate that they may provide important sources of translation machinery, or mRNAs, to the tips of axons.

## Author contributions

K-ML, BL, and JL performed the time-lapse imaging on retinal axons *in vitro*, the Netrin-1 stimulation experiments and the granule transport analyses. BL performed the cytoskeleton experiments, EB1 imaging and the Cy3-UTP worm experiments. HW conducted the *in vivo* RNA imaging experiments, performed formal analyses of data and prepared the figures. CEH supervised the project. BL, HH-WW, BT-B, JL, and CEH prepared the manuscript.

### Conflict of interest statement

The authors declare that the research was conducted in the absence of any commercial or financial relationships that could be construed as a potential conflict of interest.
