# Supplementary material for: Cue-Polarized Transport of β-actin mRNA Depends on 3′UTR and Microtubules in Live Growth Cones
Source: Front Cell Neurosci. 2018 Sep 10;12:300. doi: 10.3389/fncel.2018.00300 (PMC6139529; doi:10.3389/fncel.2018.00300)
Supplement: Supplementary file 12 [file Data_Sheet_1.PDF]

Supplementary Material

Cue-polarized transport of β-actin mRNA depends on 3’UTR and microtubules in live growth cones

Kin Mei Leung[2], Bo Lu[2], Hovy Ho-Wai Wong[1,2], Julie Qiaojin Lin, Benita Turner-Bridger and Christine E Holt*

Department of Physiology, Development and Neuroscience, University of Cambridge, Downing Street, Cambridge CB2 3DY, UK

[1]Current address: Centre of Research in Neuroscience, Brain Repair and Integrative Neuroscience Programme, Department of Neurology and Neurosurgery, The Research Institute of the McGill University Health Centre, Montreal General Hospital, Montréal, Québec H3A 2B4, Canada

[2]Authors contributed equally

***Correspondence:** Christine E Holt; ceh33@cam.ac.uk

1 Supplementary Data

Movie S1- Movie S11

2 Supplementary Figures

Figure S1- Figure S5

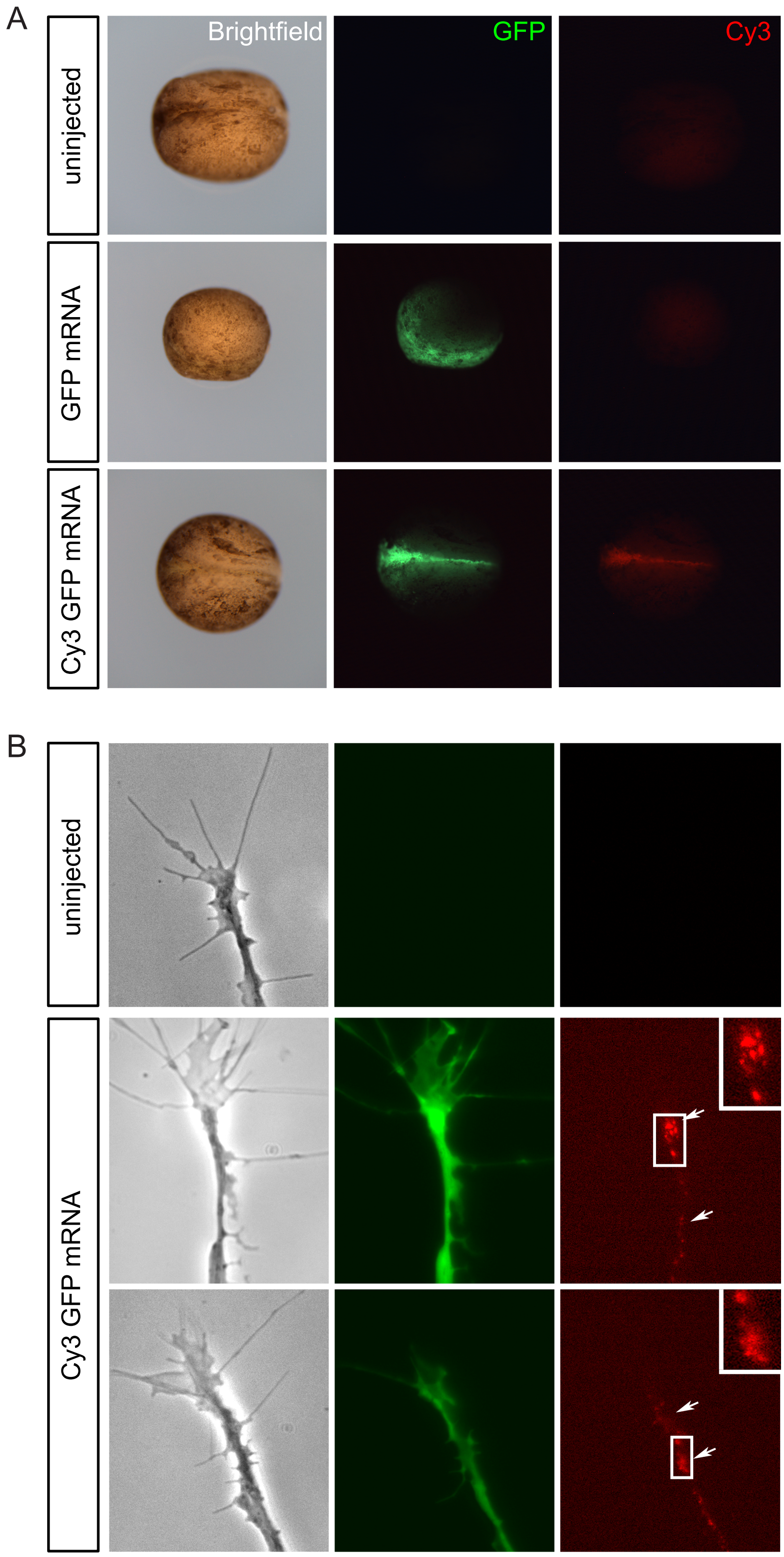

Figure S1. Related to Fig 1. Synthetic GFP mRNA injected into early dorsal blastomere was capable of being translated. (A) Embryos injected with Cy3-labelled GFP mRNA showed green fluorescence from expression of the synthetic mRNA. (B) RGC growth cones cultured from embryos injected with Cy3-labelled GFP mRNA contained both Cy3 signal indicating the presence of the synthetic mRNA and green fluorescent signal from the expression of the synthetic mRNA.

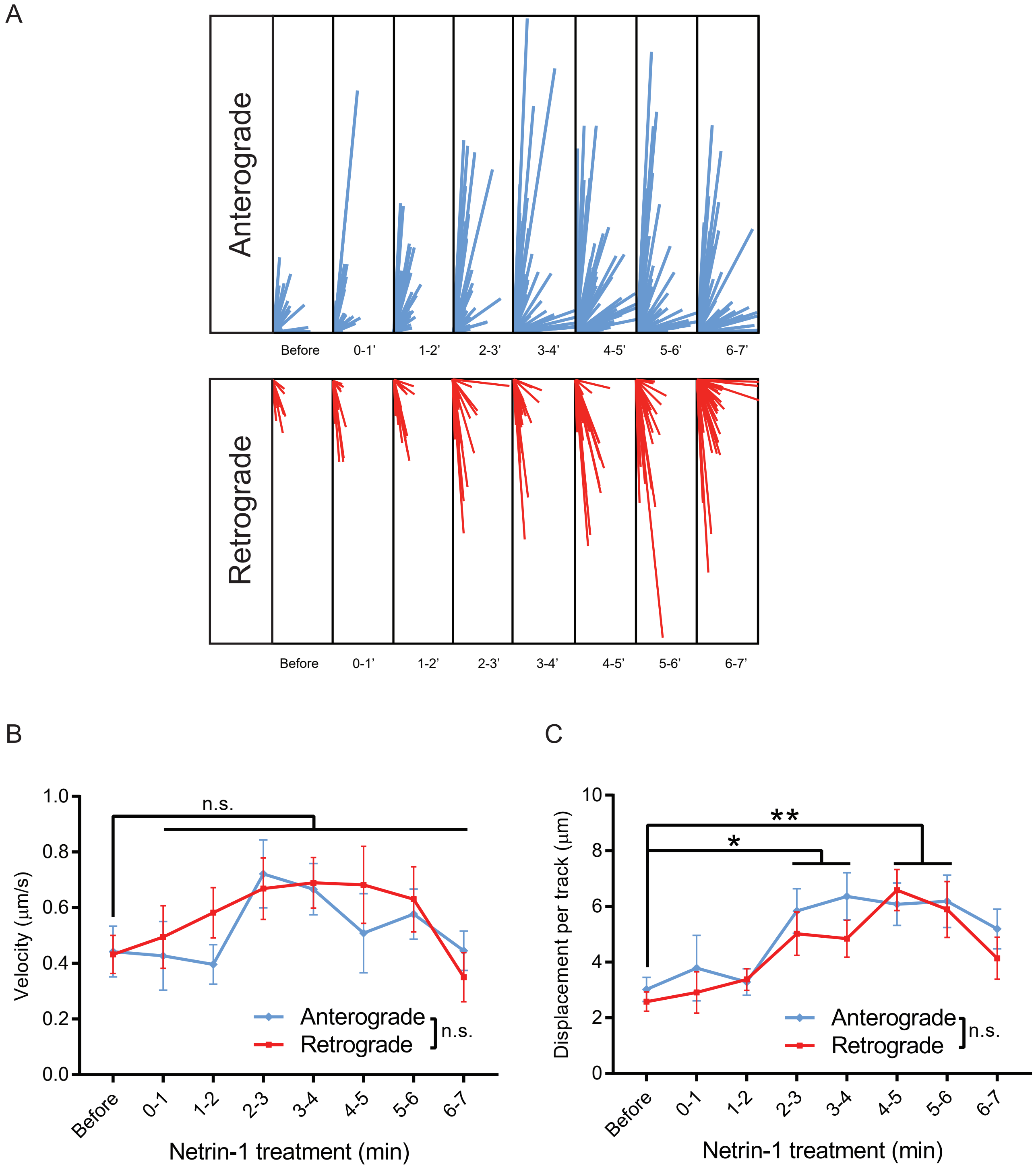

Figure S2. Related to Fig 3. Detailed analysis of β-actin mRNA granule movement in axons bath treated with netrin-1. (A) Displacement vectors of anterograde- and retrograde-moving mRNA granules. (B) The average speed remained similar between anterograde- and retrograde-moving granules before and after global netrin-1 treatment. Anterograde vs. retrograde ($F_{7, 495}$=0.512, p=0.47); Time factor, vs. before netrin-1 treatment, $^{n.s.}$p>0.30 (two-way ANOVA with Dunnett multiple-comparison test) (C) Upon global netrin-1 treatment, granule displacement per track increased in both anterograde and retrograde directions, with no significant difference between the two directions. Anterograde vs. retrograde ($F_{7, 495}$=1.492, p=0.22); Time factor, vs. before netrin-1 treatment, *p<0.05, **p<0.01 (two-way ANOVA with Dunnett multiple-comparison test)

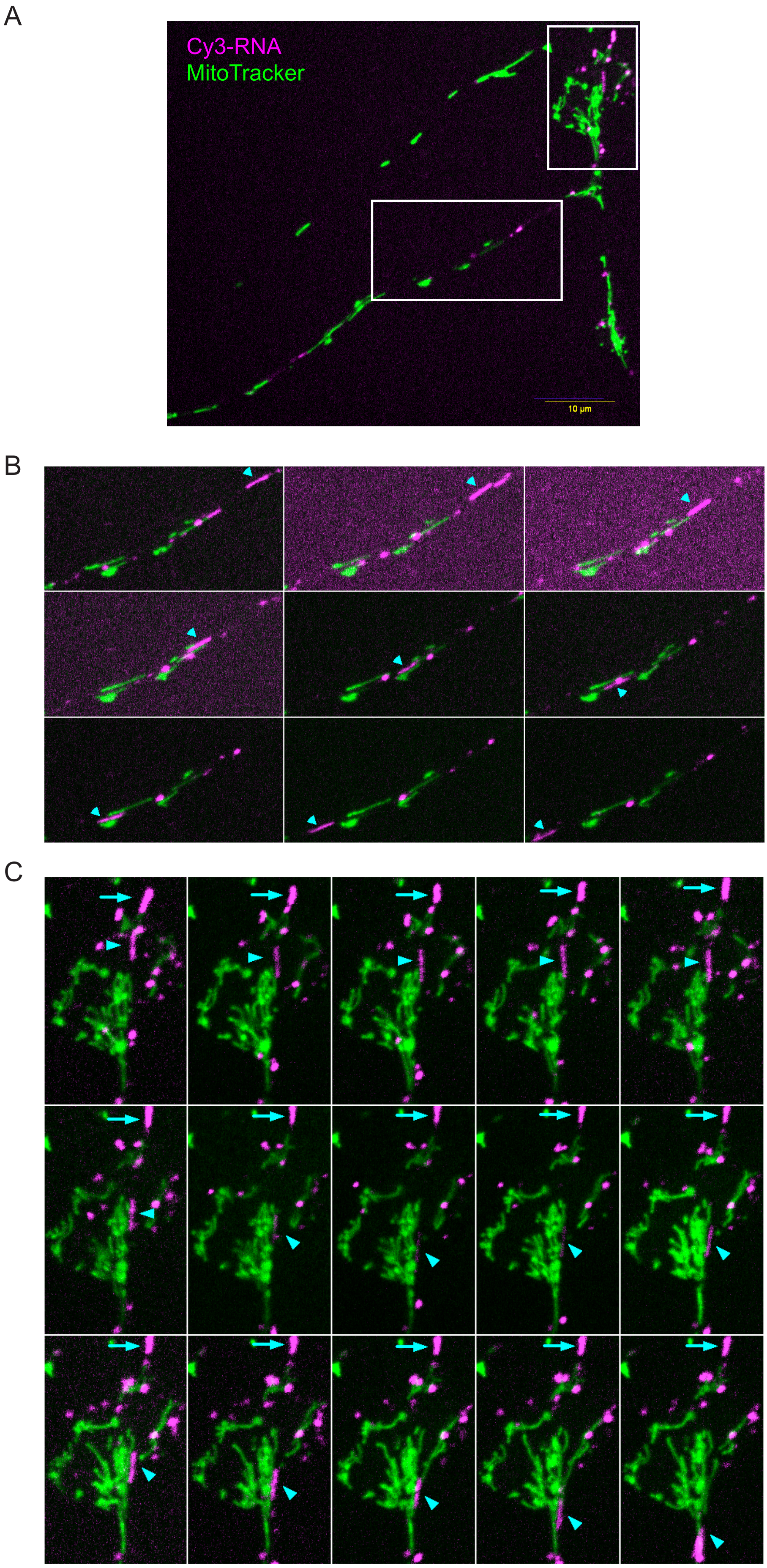

Figure S3. Related to Fig. 7. (A-C) Time-lapse images showing that RNA granules labelled by Cy3-UTP were not co-labelled by MitoTracker in axon shaft (B) and growth cone (C). Cyan arrows indicate RNA worms.

A

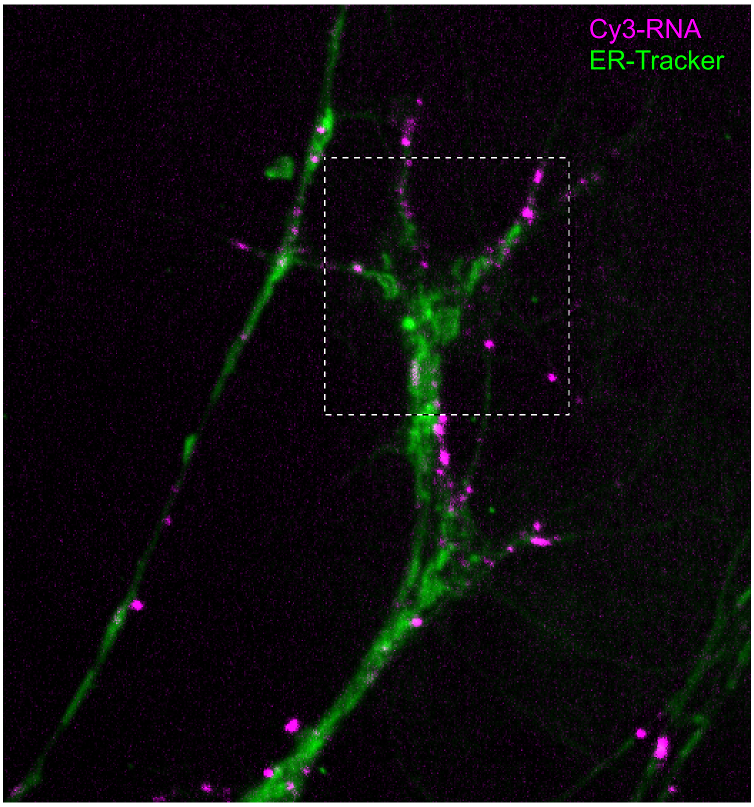

B

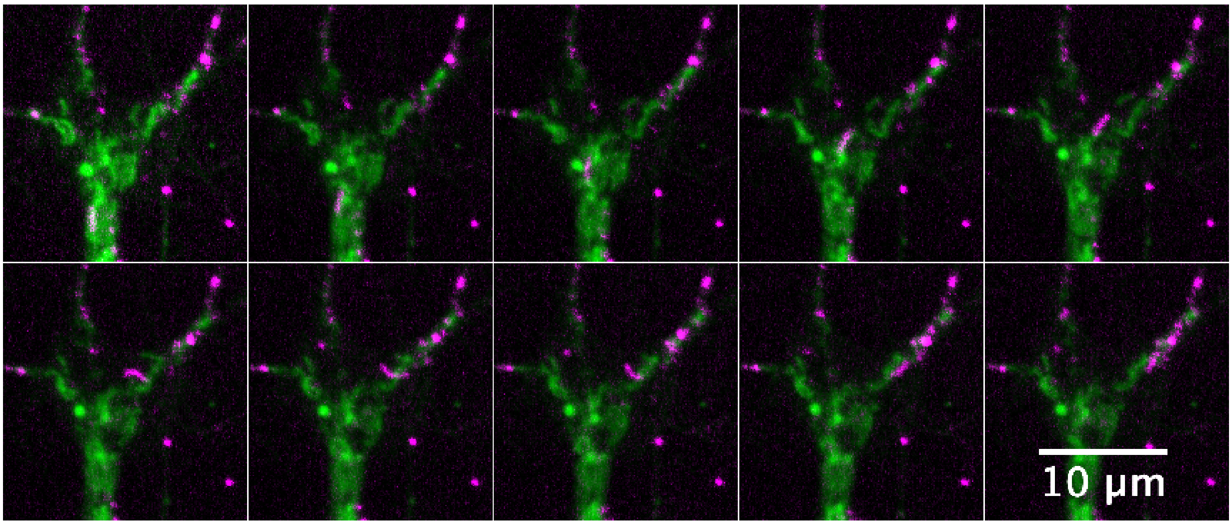

Figure S4. Related to Fig. 7. (A-B) Time-lapse images showing that RNA granules labelled by Cy3-UTP were not co-labelled by ER-Tracker.

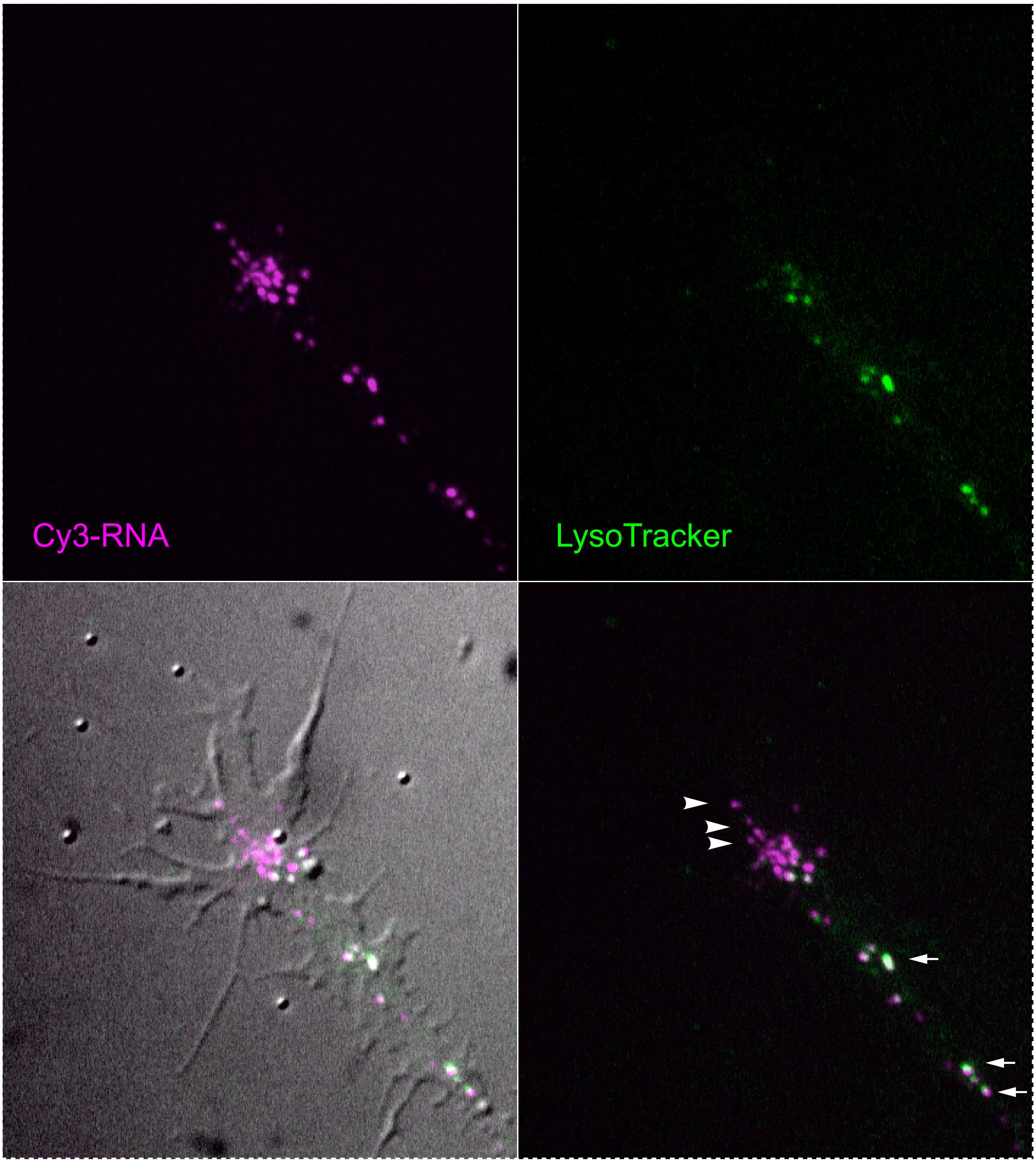

Figure S5. Related to Fig. 7. A small population of RNA granules labelled by Cy3-UTP were co-labelled by LysoTracker (arrows). Arrowheads indicate peripherally-localized Cy3-RNA granules and are free of apparent LysoTracker staining.
